# Quantum Tunneling-Induced Membrane Depolarization Can Explain the Cellular Effects Mediated by Lithium: Mathematical Modeling and Hypothesis

**DOI:** 10.3390/membranes11110851

**Published:** 2021-11-01

**Authors:** Lubna Khreesha, Abdallah Barjas Qaswal, Baheth Al Omari, Moath Ahmad Albliwi, Omar Ababneh, Ahmad Albanna, Abdelrahman Abunab’ah, Mohammad Iswaid, Salameh Alarood, Hasan Guzu, Ghadeer Alshawabkeh, Fuad Mohammed Zayed, Mohammad Awad Abuhilaleh, Mohammad Nayel Al-Jbour, Salameh Obeidat, Aiman Suleiman

**Affiliations:** 1 School of Medicine, The University of Jordan, Amman 11942, Jordan; bomari81@gmail.com (B.A.O.); moathelbalawe2018@yahoo.com (M.A.A.); omar.ababneh@ju.edu.jo (O.A.); Dr.albanna10@gmail.com (A.A.); abd93abunabah@yahoo.com (A.A.); Mohd_Iswaid@hotmail.com (M.I.); Sal.habashneh@gmail.com (S.A.); Fuad.41994@gmail.com (F.M.Z.); Mohd_jbour@yahoo.com (M.N.A.-J.); 2 Anesthesia Department, Farah Medical Campus, 18 Mai Zeyadeh Street, Amman 11942, Jordan; hasangu91@hotmail.com; 3 Anesthesia and Pain Management Department, King Hussein Cancer Center, Amman 11942, Jordan; shawabkehgk@gmail.com; 4 School of Medicine, University of Science and Technology, Sana’a 13064-15201, Yemen; Mohammmed.krishan@gmail.com; 5 Department of Anesthesia, Intensive Care and Pain Management, Harvard Medical School, Beth Israel Deaconess Medical Center, Boston, MA 02215, USA; sobeidat@bidmc.harvard.edu (S.O.); asuleima@bidmc.harvard.edu (A.S.)

**Keywords:** quantum tunneling, lithium, quantum biology, voltage-gated channel, quantum conductance, depolarization, GSK-3*β*, wnt/β-catenin

## Abstract

Lithium imposes several cellular effects allegedly through multiple physiological mechanisms. Membrane depolarization is a potential unifying concept of these mechanisms. Multiple inherent imperfections of classical electrophysiology limit its ability to fully explain the depolarizing effect of lithium ions; these include incapacity to explain the high resting permeability of lithium ions, the degree of depolarization with extracellular lithium concentration, depolarization at low therapeutic concentration, or the differences between the two lithium isotopes Li-6 and Li-7 in terms of depolarization. In this study, we implemented a mathematical model that explains the quantum tunneling of lithium ions through the closed gates of voltage-gated sodium channels as a conclusive approach that decodes the depolarizing action of lithium. Additionally, we compared our model to the classical model available and reported the differences. Our results showed that lithium can achieve high quantum membrane conductance at the resting state, which leads to significant depolarization. The quantum model infers that quantum membrane conductance of lithium ions emerges from quantum tunneling of lithium through the closed gates of sodium channels. It also differentiates between the two lithium isotopes (Li-6 and Li-7) in terms of depolarization compared with the previous classical model. Moreover, our study listed many examples of the cellular effects of lithium and membrane depolarization to show similarity and consistency with model predictions. In conclusion, the study suggests that lithium mediates its multiple cellular effects through membrane depolarization, and this can be comprehensively explained by the quantum tunneling model of lithium ions.

## 1. Introduction

Lithium ions possess the ability to depolarize the resting membrane potential [1,2,3,4,5]. On the other hand, lithium has diverse cellular effects that cannot be unified under one trigger or mechanism, such as neuroprotection [6], immunomodulation [7], proliferative and anti-proliferative effects [8,9], wound healing [10,11], stem cells proliferation and differentiation [12,13], and pigmentation [14,15]. Interestingly, depolarized resting membrane potential imposes similar cellular effects as those mediated by lithium [16]. Hence, an association between membrane depolarization and cellular effects of lithium can be hypothesized.

However, the depolarization of resting potential mediated by lithium ions is challenging and cannot be fully understood by classical electrophysiology alone. This issue is attributed to the following reasons: (1) The resting membrane potential is determined mainly by sodium and potassium ions [17,18], and if lithium ions are added, it is expected that lithium will have the same low permeability of sodium ions at the resting state [2,19,20] because sodium channels are selective for sodium and lithium ions to the same degree approximately and the permeability ratio between them is near to 1 [21], while potassium channels have low selectivity for both sodium and lithium ions [22]. This low permeability is conceptually incompatible with the experimental results that showed lithium has a high resting permeability that can induce remarkable depolarization [1]; (2) The expected low lithium permeability at the resting state cannot explain the experimentally observed correlation between the degree of depolarization and extracellular lithium concentration [1], nor does it explain the hypothesized depolarization effect induced by lithium at its therapeutic concentrations (0.6–1.2 mEq/L) because these low concentrations of lithium along with its low permeability cannot affect the membrane potential if the Goldman–Hodgkin–Katz (GHK) equation is applied [3,4,5]; (3) The differential effects on membrane potential between the two lithium isotopes cannot be explained classically because the permeability ratio between Li-6/Li-7 according to their diffusion is expected to be 1.006 [23], which is critically low and shows no significant difference in membrane depolarization if GHK equation was applied.

Hence, we hypothesize that the membrane depolarization induced by lithium ions is due to high resting permeability that cannot be explained classically for the previously mentioned reasons.

The model of quantum tunneling of ions [24] used in this study addresses these three challenges and aids in better understanding of the depolarization effect mediated by lithium. The previously mentioned three challenges will be further elaborated in the following sections to show how the quantum model can solve these challenges and give a more comprehensive picture of the electrophysiological features of lithium ions. 

Accordingly, the study aims to show that membrane depolarization induced by lithium ions cannot be fully explained by the classical electrophysiology alone and that quantum modelling gives a reasonable mechanism that can bring the pieces of the puzzle together. We hypothesize an association between membrane depolarization and the cellular effects of lithium by shedding light on the similarity and consistency between the effects of lithium ions and the effects of membrane depolarization.

To the authors’ knowledge, this article represents the first attempt to unify the cellular effects of lithium under one main trigger, which is membrane depolarization. Throughout the paper, when we say ‘high resting permeability of lithium ions’, we refer to the gradual (10s of mins or hs) intracellular accumulation of lithium and depolarization that occurs when spontaneously active tissues are bathed with high lithium concentrations.

## 2. The Mathematical Modeling of Quantum Tunneling of Lithium Ions

The model of quantum tunneling of ions states that ions have non-zero probability to pass through closed channels, including closed voltage-gated channels [24]. These closed channels form an energy barrier, which is higher than the kinetic energy of the passing ions. The model suggests a new perspective on ion transport in the biological systems, and this transport can be called quantum transport via quantum tunneling. The unique aspect of this kind of transport is that it is not necessary for the energy of the barrier to be less than the energy of the passing particle for the transport to happen. This model is emerging from one important consequence of quantum mechanics, which is quantum tunneling. This phenomenon can be mathematically represented by solving the Schrodinger equation and applying Born’s rule to find the probability of tunneling [25].

The quantum tunneling probability through an energy barrier can be calculated by solving the Schrödinger equation and applying Born’s rule [25]:

(1) $$T_{Q}=e^{\frac{-\sqrt{8m}}{\hbar }\int_{X1}^{X2}\sqrt{U\left(x\right)-KE}dx}$$

where *T_Q_* is the tunneling probability, *m* is the mass of ion, ℏ is the reduced Planck constant ($1.05\times 10^{-34}$ Js), *U(x)* is the energy of barrier with respect to the ion’s position *x*, *KE* is the kinetic energy of ion, and *X*1*-X*2 is the forbidden region where the ion cannot pass.

Equation (1) has been applied on the closed gate of voltage-gated channels to explore the quantum conductance of these channels [26,27]. The derivation of the final form of the tunneling probability of ions through the closed gates has been justified previously [26,27]. The quantum tunneling model of lithium ions is applied on voltage-gated sodium channels since they are selective for lithium and sodium ions by the same degree. 

The final form of the tunneling probability for ions through the closed gate of voltage-gated sodium channels can be calculated by the following equation [26,27]:

(2) $$T_{Q}{}_{\left(Li\right)}=e^{\frac{-\sqrt{8m}}{\hbar }\times \frac{2w}{3g}\sqrt{{\left(g-KE\right)}^{3}}}$$

where *T_Q_* is the tunneling probability, *m* is the mass of lithium ion, ℏ is the reduced Planck constant, *w* is the length of gate, *g* is the energy of gate which represents the energy required for the ion to pass the closed gate, and *KE* is the kinetic energy of lithium ion.

Here, we provide the derivation of the final form of the equation of tunneling probability to make it more understandable. This equation has been derived before [26,27].

The barrier energy of closed gate U(x) can be illustrated as a regular electric field that resists the movement of ions [24,26,27]. Therefore, U(x) can be calculated by the following equation:

(3) $$U\left(x\right)=\frac{g}{w}x$$

Equation (3) means that the energy required to open the closed gate is divided regularly over the gate length. Accordingly, the integral in Equation (1) can be written and solved as in the following form:

(4) $$\int_{X1}^{X2}\sqrt{\frac{g}{w}x-KE}dx=\frac{2w}{3g}\sqrt{{\left(\frac{g}{w}x_{2}-KE\right)}^{3}}-\frac{2w}{3g}\sqrt{{\left(\frac{g}{w}x_{1}-KE\right)}^{3}}$$

$x_{2}$ is at the end of the length of gate and this means $x_{2}=w$, while $x_{1}$ is where $\frac{g}{w}x_{1}=KE$. So, the region between $x_{1}$ and $x_{2}$ is the forbidden region where ion can not pass.

Accordingly, the integral can be written as in the following form:

(5) $$\int_{X1}^{X2}\sqrt{\frac{g}{w}x-KE}dx=\frac{2w}{3g}\sqrt{{\left(g-KE\right)}^{3}}$$

Eventually, the solution of the integral can be substituted in Equation (1) to obtain Equation (2).

**Figure 1:**
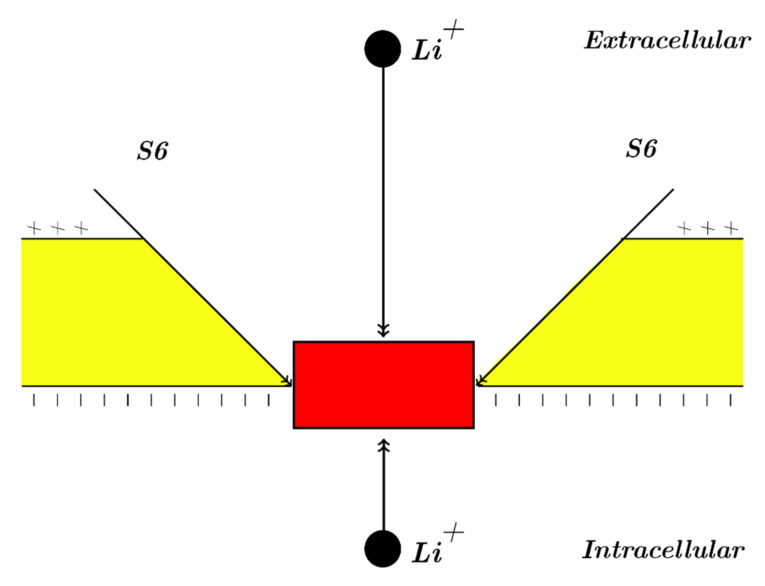
The figure represents a schematic diagram of the closed intracellular gate, which is red in color, made by a constriction of the hydrophobic residues of the S6 alpha helices of the voltage-gated sodium channel. The extracellular lithium ions pass across the membrane voltage until hitting the closed gate, while the intracellular lithium ions hit the closed gate before going through the membrane voltage.

Our study will focus mainly on the resting membrane potential, in which voltage-gated sodium channels are closed by forming a hydrophobic gate or by a constriction at the intracellular end of cells. Accordingly, our modeling will be applied on the closed intracellular hydrophobic gate [24,26,27]. As a result, extracellular lithium ions will pass through the membrane voltage *V_m_* until hitting the closed intracellular gate, attaining a kinetic energy that equals to *qV_m_*; these ions will also obtain an average thermal kinetic energy of $\frac{3}{2}K_{B}T=0.64\times 10^{-20}$ J [26,27]. On the other hand, intracellular lithium ions will hit the intracellular gate having only the average thermal kinetic energy of $\frac{3}{2}K_{B}T=0.64\times 10^{-20}$ J [26,27]. See Figure 1. The voltage across the closed gate can be neglected because its length is short relative to whole membrane thickness; hence, it is assumed that extracellular lithium ions go across the whole membrane voltage *V_m_.*

Consequently, the tunneling probability of extracellular lithium ions can be calculated by the following equation:

(6) $$T_{Q}{}_{\left(Li\right)E}=e^{\frac{-\sqrt{8m}}{\hbar }\times \frac{2w}{3g}\sqrt{{\left(g-(qV_{m}+\frac{3}{2}K_{B}T)\right)}^{3}}}$$

where $T_{Q}{}_{\left(Li\right)E}$ is the tunneling probability of extracellular lithium ions, *q* is the charge of lithium ions ($1.6\times 10^{-19}$ C), *V_m_* is the resting membrane potential, *K_B_* is the Boltzmann’s constant ($1.38\times 10^{-23}$ J/K), and *T* is the body temperature (310 K). The membrane potential *V_m_* represents an absolute value so that the kinetic energy of extracellular lithium ions $qV_{m}$ will be a positive value. Therefore, the equations and the graphs in the upcoming sections will deal with absolute values of membrane potential, which is negative inside with regard to outside.

The tunneling probability of intracellular lithium ions can be calculated by the following equation:

(7) $$T_{Q}{{}_{\left(Li\right)}}_{I}=e^{\frac{-\sqrt{8m}}{\hbar }\times \frac{2w}{3g}\sqrt{{\left(g-\left(\frac{3}{2}K_{B}T\right)\right)}^{3}}}$$

where $T_{Q}{{}_{\left(Li\right)}}_{I}$ is the tunneling probability of intracellular lithium ions.

As a result of quantum tunneling of lithium ions through the closed gate of channels, these channels will have a conductance called quantum conductance. Therefore, the quantum conductance of single channel for lithium ions $C_{Q}{}_{\left(Li\right)}$ [25,28]:

(8) $$C_{Q}{}_{\left(Li\right)}=\frac{q^{2}}{h}T_{Q\left(Li\right)}$$

where *q* is the charge of lithium ion ($1.6\times 10^{-19}$ C), *h* is the Planck constant ($6.6\times 10^{-34}$ Js), and $T_{Q\left(Li\right)}$ is the tunneling probability of lithium ions. The unit of quantum conductance of single channel is Siemens (S).

Consequently, the cellular membrane with a certain number of channels will have quantum membrane conductance. Hence, the quantum membrane conductance of lithium ions $MC_{Q}{}_{\left(Li\right)}$ at certain channels density *D* (channels/cm^2^) [17,18]:

(9) $$MC_{Q}{}_{\left(Li\right)}=D\times C_{Q}{}_{\left(Li\right)}$$

The unit of quantum membrane conductance that will be used in this study is mS/cm^2^.

To assess the influence of quantum tunneling of ions and its associated quantum conductance on the resting membrane potential, the Goldman–Hodgkin–Katz (GHK) equation will be used. In the present study, two versions of the GHK equation will be used: (1) The classical version which evaluates the influence of classical transport of lithium ions through open channels. This transport is indicated by the leak membrane conductance of lithium ions $MC_{Li}$, which is almost equivalent to the leak membrane conductance of sodium ions $MC_{Na}$. (2) The quantum version evaluates the influence of quantum transport of lithium ions via quantum tunneling through the closed sodium channels.

The classical version of GHK equation [17,18]:

(10) $$[K{]}_{E}\left(MC_{K}\right)+[Na{]}_{E}\left(MC_{Na}\right)+{\left[Li\right]}_{E}\left(MC_{Li}\right)=e^{\frac{-FV_{m}}{RT}}{\left([K{]}_{I}\left(MC_{K}\right)+[Na\right]}_{I}\left(MC_{Na}\right)+\left[Li{]}_{I}\left(MC_{Li}\right)\right)$$

where [] is the concentration, *K* refers to potassium ions, *Na* refers to sodium ions, *Li* refers to lithium ions, *E* refers to extracellular, *I* refers to intracellular, $MC_{K}$ is the leak membrane conductance of potassium ions at the resting state, $MC_{Na}$ is the leak membrane conductance of sodium ions at the resting state, $MC_{Li}$ is the leak membrane conductance of lithium ions at the resting state, *F* is the Faraday constant (96,485.33 C/mol), *V_m_* is the resting membrane potential, *R* is the gas constant (8.31 J/Kmol), and *T* is the body temperature in Kelvin (310 K). The negative sign is added to the term $\frac{FV_{m}}{RT}$ to obtain an absolute value of the membrane potential, which is negative inside with regard to outside.

The quantum version of GHK equation [26,27] that will be used to evaluate the influence of quantum tunneling of ions on the resting membrane potential:

(11) $$[K{]}_{E}\left(MC_{K}\right)+[Na{]}_{E}\left(MC_{Na}\right)+{\left[Li\right]}_{E}\left(MC_{Q\left(Li\right)E}\right)=e^{\frac{-FV_{m}}{RT}}{\left([K{]}_{I}\left(MC_{K}\right)+[Na\right]}_{I}\left(MC_{Na}\right)+\left[Li{]}_{I}\left(MC_{Q\left(Li\right)I}\right)\right)$$

where $MC_{Q\left(Li\right)E}$ is the quantum membrane conductance of extracellular lithium ions and $MC_{Q\left(Li\right)I}$ is the quantum membrane conductance of intracellular lithium ions. The negative sign is added to the term $\frac{FV_{m}}{RT}$ to obtain an absolute value of membrane potential.

In this study, the following values will be considered as reference values to calculate the resting membrane potential without adding lithium ions: ${\left[K\right]}_{E}=4$ mmol/L [17,18],${\left[K\right]}_{I}=140$ mmol/L [17,18], ${\left[Na\right]}_{E}=142$ mmol/L [17,18], ${\left[Na\right]}_{I}=14$ mmol/L [17,18], $MC_{Na}=0.005$ mS/cm^2^ [17,18], $MC_{K}=0.5$ mS/cm^2^ [17,18]. These values yield a membrane potential V_m_= 0.087 V by applying the classical version of GHK equation. We chose this value of membrane potential to show graphically and numerically the results after adding lithium ions as in the following sections. However, any value of membrane potential, as long as it is negative inside with regard to outside, can be used to assess the influence of classical transport and quantum transport of lithium ions on the resting membrane potential.

The mathematical model of quantum tunneling in the present study is an extension of a previous model used before [24]. However, in the present study, the model is utilized more beneficially to investigate the mass isotopic effect of lithium ions and to assess how other factors such as the energy of gate, the length of gate, the membrane potential, and the density of channels affect the values of quantum tunneling probability and quantum conductance of lithium mathematically and graphically. This approach allowed a wide range of values to be applied in the model for a better comparison between the two isotopes of lithium.

## 3. Results

In this section, the quantum tunneling probability, the quantum conductance of a single channel, and the quantum membrane conductance will be plotted according to different factors for the two lithium isotopes Li-6 and Li-7. These plots are important to understand the depolarization effect of lithium ions and to account for the differential effects of the two lithium isotopes depending on the degree of membrane depolarization. Additionally, the resting membrane potential is evaluated under the influence of classical transport of lithium ions and quantum tunneling of lithium ions to show the difference in the degree of depolarization between the classical and quantum transport and also between the two isotopes in the two types of transport. The graphs will be semi-log graphs, especially for quantum tunneling probability, quantum conductance of single channel, and quantum membrane conductance.

The mass of Li-7 is $1.15\times 10^{-26}$ Kg, and the mass of Li-6 is $9.83\times 10^{-27}$ Kg (less than Li-7 by the mass of one neutron). Moreover, the length of the intracellular gate will be over the range $\left(0-2\right)\times 10^{-10}$ m to explore how the quantum variables behave with respect to the length of gate. This range is reasonable because the intracellular gate is made by a constriction of four hydrophobic amino acids at the same level [29]. Hence, the length of gate should be equal to the length of a single amino acid, which is $1.5\times 10^{-10}$ m [30]. However, considering the tilt angle of the S6 alpha helix with the cellular membrane [31], the length of gate may be reduced to $0.5\times 10^{-10}$ m [24,32]. Therefore, we take the previously mentioned range to cover all possible values including the extreme ones with an average value $1\times 10^{-10}$ m. In addition, the energy of gate *g* can be estimated by considering this equation $q_{gate}\left(V_{1/2}-V_{m}\right)$, which represents the energy required to open the closed channel at certain membrane potential, hence, it can be used as an estimation value for the barrier energy of the closed gate [26,27]. This value depends on the moving gating charge $q_{gate}$ to open the closed gate, the half-activation voltage V_1/2_ at which half of the channels are open, and the membrane potential at a given moment *V_m_*. *V_m_* = 0.087 V will be chosen as the reference value to estimate the energy of gate *g* since it represents the initial resting membrane potential. However, the value of *g* varies according to the type of tissue and the type of sodium channel. For example, the voltage-gated sodium channels in the cardiac tissue Nav1.5 have $q_{gate}=3.8e$ [33] and V_1/2_= 0.0326 V [34]. Thus,$\;g=3.31\times 10^{-20}$ J, and if $q_{gate}=5e$ [34], $g=4.35\times 10^{-20}$ J. Additionally, the gating charge of sodium channels Nav1.2 in the neuronal membrane $q_{gate}=9.2e$ [35] and the half-activation voltage is V_1/2_ = 0.043 V [36,37]. Thus, $g=6.48\times 10^{-20}$ J. Therefore, to account for the variability in the *g* values according to the types of tissues and channels, we take the range $\left(3-7\right)\times 10^{-20}$ J for investigation with an average value $5\times 10^{-20}$ J. Furthermore, the density of sodium channels *D* can reach up to $10^{11}$ channels/cm^2^ [17].

### 3.1. The Quantum Tunneling Probability of Lithium Isotopes

The tunneling probability of extracellular lithium isotopes:

(12) $$T_{Q\left(Li-7\right)E}=e^{-19.2\frac{L}{G}\sqrt{{\left(G-16V_{m}-0.64\right)}^{3}}}$$

(13) $$T_{Q\left(Li-6\right)E}=e^{-17.8\frac{L}{G}\sqrt{{\left(G-16V_{m}-0.64\right)}^{3}}}$$

The Equations (12) and (13) come after substituting the values of constants in Equation (6), taking into consideration that $L=\frac{w}{10^{-10}}$ (and multiplying the exponent by $10^{-10}$), and $G=\frac{g}{10^{-20}}$ (and dividing the exponent by $10^{-20}$), and taking $10^{-20}$ as a common factor from the square root and multiplying the exponent by $\sqrt{{\left(10^{-20}\right)}^{3}}=10^{-30}$. This simplifies the equations and makes it easy to deal with numerical values. This is applied on all of the following equations. Moreover, the number 19.2 in Equation (12) is a result of the following calculations: $\frac{\sqrt{8\times 1.15\times 10^{-26}}}{1.05\times 10^{-34}}\times \frac{2\times 10^{-10}\times 10^{-30}}{3\times 10^{-20}}=19.2$ and the number 17.8 in Equation (13) is a result of the following calculations: $\frac{\sqrt{8\times 9.83\times 10^{-27}}}{1.05\times 10^{-34}}\times \frac{2\times 10^{-10}\times 10^{-30}}{3\times 10^{-20}}=17.8$.

According to these equations, $L=\frac{w}{10^{-10}}$ and $=\frac{g}{10^{-20}}$; when we say for example that L = 2 m, this means that the actual length w is $2\times 10^{-10}$ m and when we say G=7 J, this means that the actual energy g is $7\times 10^{-20}$ J. This approach simplifies the numbers and makes it easier to deal with them.

Moreover, in the following graphs, we evaluate the quantum variables according to a range of values of the gate’s energy *G*, the length of the gate *L*, the membrane potential *V_m_*, and the density of channels *D*. However, when we evaluate according to one of these variables, we set the others as G=5 J, L=1 m, $V_{m}=0.087$ V, and $D=10^{11}$ channels/cm^2^ to be substituted in the equations.

**Figure 2:**
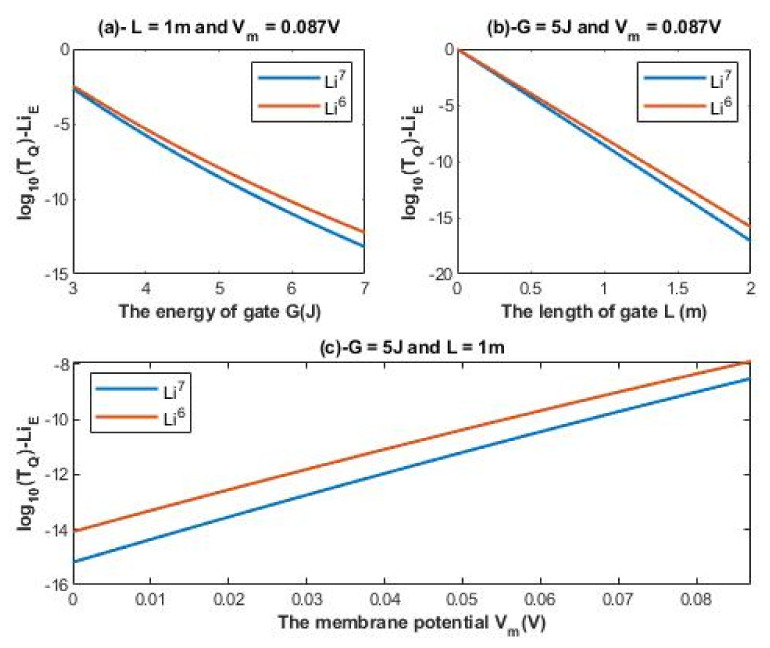
(**a**): The figure represents the relationship between the common logarithm of tunneling probability of the extracellular lithium isotopes $\log_{10}(T_{Q})-Li_{E}$ and the energy of the gate *G* by setting L=1 m and $V_{m}=0.087$ V and across the range of *G* from 3 J to 7 J. (**b**): The figure represents the relationship between the common logarithm of tunneling probability of the extracellular lithium isotopes $\log_{10}(T_{Q})-Li_{E}$ and the length of the gate *L* by setting G=5 m and $V_{m}=0.087$ V and across the range of *L* from 0 m to 2 m. (**c**): The figure represents the relationship between the common logarithm of tunneling probability of the extracellular lithium isotopes $\log_{10}(T_{Q})-Li_{E}$ and the membrane potential by setting G=5  J and L=1  m and across the range of membrane potential from 0 V to 0.087 V.

According to Equations (12) and (13), the relationship between the common logarithm of tunneling probability of extracellular lithium isotopes $\log_{10}(T_{Q})-Li_{E}$ and the energy of the gate *G*, the length of the gate *L*, and the membrane potential *V_m_* can be evaluated. See Figure 2.

The tunneling probability of intracellular lithium isotopes:


(14) $$T_{Q\left(Li-7\right)I}=e^{-19.2\frac{L}{G}\sqrt{{\left(G-0.64\right)}^{3}}}$$

(15) $$T_{Q\left(Li-6\right)I}=e^{-17.8\frac{L}{G}\sqrt{{\left(G-0.64\right)}^{3}}}$$

**Figure 3:**
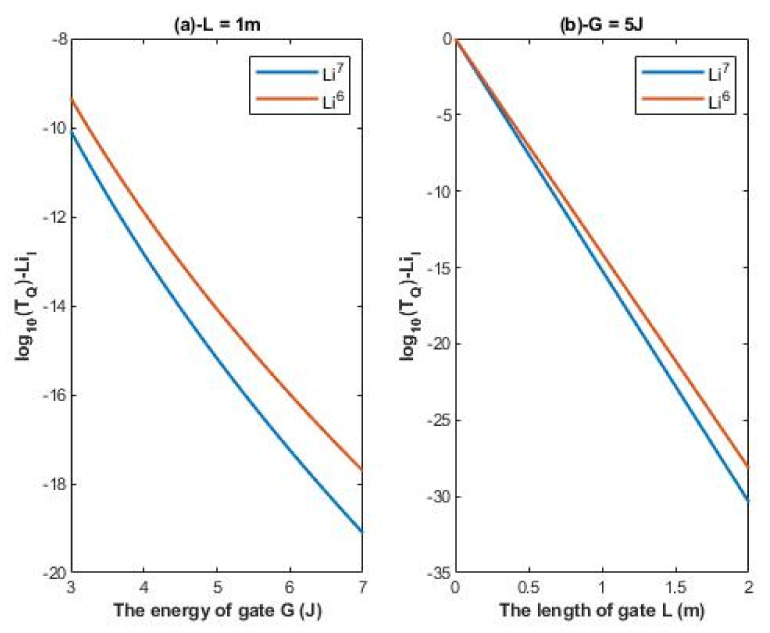
(**a**): The figure represents the relationship between the common logarithm of tunneling probability of the intracellular lithium isotopes $\log_{10}(T_{Q})-Li_{I}$ and the energy of gate *G* by setting L=1 m and across the range of *G* from 3 J to 7 J. (**b**): The figure represents the relationship between the common logarithm of tunneling probability of the intracellular lithium isotopes $\log_{10}(T_{Q})-Li_{I}$ and the length of the gate *L* by setting G=5 J and across the range of *L* from 0 m to 2 m.

According to Equations (14) and (15), the relationship between the common logarithm of quantum tunneling probability of intracellular lithium isotopes $\log_{10}(T_{Q})-Li_{I}$ and the energy of the gate *G* and the length of the gate *L* can be evaluated. See Figure 3.

### 3.2. The Quantum Conductance of Single Channel for Lithium Isotopes

By substituting the constants in Equation (8), the quantum conductance of single channel for extracellular lithium isotopes:


(16) $$C_{Q\left(Li-7\right)E}=3.88\times 10^{-5}e^{-19.2\frac{L}{G}\sqrt{{\left(G-16V_{m}-0.64\right)}^{3}}}$$

(17) $$C_{Q\left(Li-6\right)E}=3.88\times 10^{-5}e^{-17.8\frac{L}{G}\sqrt{{\left(G-16V_{m}-0.64\right)}^{3}}}$$

The unit of quantum conductance of a single channel is Siemens (S) and the number $3.88\times 10^{-5}$ comes from the following calculation $\frac{q^{2}}{h}=\frac{{\left(1.6\times 10^{-19}\right)}^{2}}{6.6\times 10^{-34}}=3.88\times 10^{-5}$.

**Figure 4:**
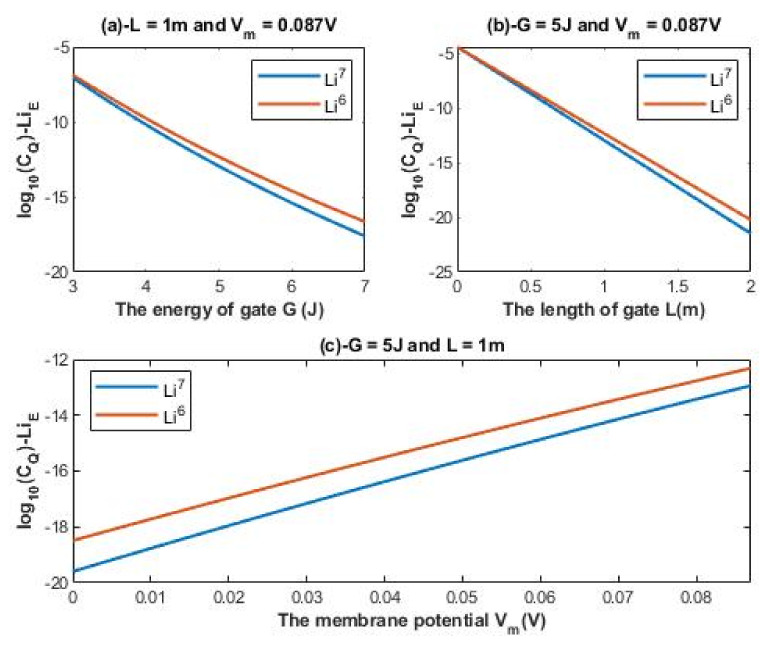
(**a**): The figure represents the relationship between the common logarithm of quantum conductance of single channel for the extracellular lithium isotopes $\log_{10}(C_{Q})-Li_{E}$ and the energy of gate *G* by setting L=1 m and $V_{m}=0.087$ V and across the range of *G* from 3 J to 7 J. (**b**): The figure represents the relationship between the common logarithm of quantum conductance of single channel for the extracellular lithium isotopes $\log_{10}(C_{Q})-Li_{E}$ and the length of the gate *L* by setting G=5 J and $V_{m}=0.087$ V and across the range of L from 0 m to 2 m. (**c**): The figure represents the relationship between the common logarithm of quantum conductance of single channel for the extracellular lithium isotopes $\log_{10}(C_{Q})-Li_{E}$ and the membrane potential by setting G=5 J and L=1 m and across the range of membrane potential from 0 V to 0.087 V.

According to Equations (16) and (17), the relationship between the common logarithm of quantum conductance of single channel for extracellular lithium isotopes $\log_{10}(C_{Q})-Li_{E}$ and the energy of the gate *G*, the length of the gate *L*, and the membrane potential *V_m_* can be evaluated. See Figure 4.

The quantum conductance of single channel for intracellular lithium isotopes:


(18) $$C_{Q\left(Li-7\right)I}=3.88\times 10^{-5}e^{-19.2\frac{L}{G}\sqrt{{\left(G-0.64\right)}^{3}}}$$

(19) $$C_{Q\left(Li-6\right)I}=3.88\times 10^{-5}e^{-17.8\frac{L}{G}\sqrt{{\left(G-0.64\right)}^{3}}}$$

The unit of quantum conductance of single channels is Siemens (S).

**Figure 5:**
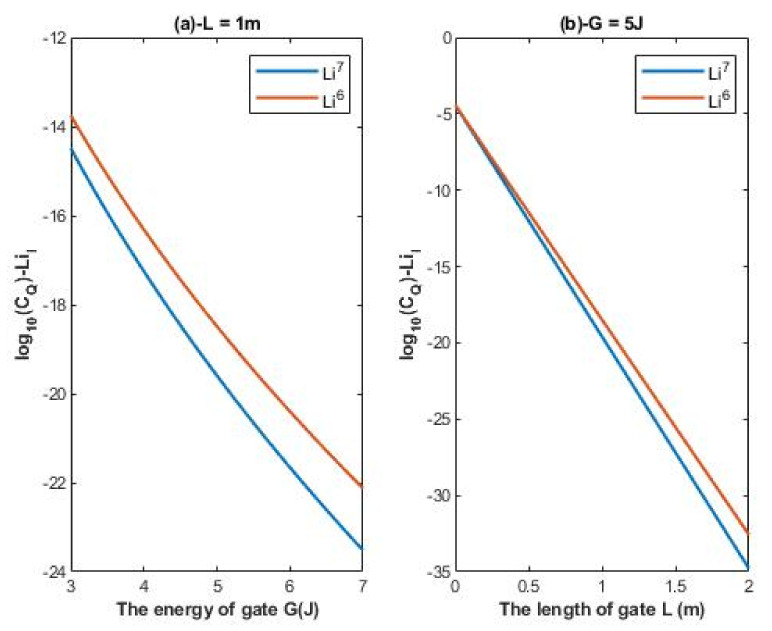
(**a**): The figure represents the relationship between the common logarithm of quantum conductance of single channel for the intracellular lithium isotopes $\log_{10}(C_{Q})-Li_{I}$ and the energy of gate *G* by setting L=1 m and across the range of *G* from 3 J to 7 J. (**b**): The figure represents the relationship between the common logarithm of quantum conductance of single channel for the intracellular lithium isotopes $\log_{10}(C_{Q})-Li_{I}$ and the length of the gate *L* by setting G=5 J and across the range of *L* from 0 m to 2 m.

According to Equations (18) and (19), the relationship between the common logarithm of quantum conductance of single channel for intracellular lithium isotopes $log_{10}(C_{Q})-Li_{I}$ and the energy of the gate *G* and the length of the gate *L* can be evaluated. See Figure 5.

### 3.3. The Quantum Membrane Conductance of Lithium Isotopes

The quantum membrane conductance of extracellular lithium isotopes:


(20) $$MC_{Q\left(Li-7\right)E}=3.88\times 10^{-2}e^{-19.2\frac{L}{G}\sqrt{{\left(G-16V_{m}-0.64\right)}^{3}}}\times D$$

(21) $$MC_{Q\left(Li-6\right)E}=3.88\times 10^{-2}e^{-17.8\frac{L}{G}\sqrt{{\left(G-16V_{m}-0.64\right)}^{3}}}\times D$$

The unit of quantum membrane conductance is mS/cm^2^ and the number $3.88\times 10^{-5}$ is converted to $3.88\times 10^{-2}$ by multiplying by $10^{3}$ to convert the unit from (S) to (mS).

**Figure 6:**
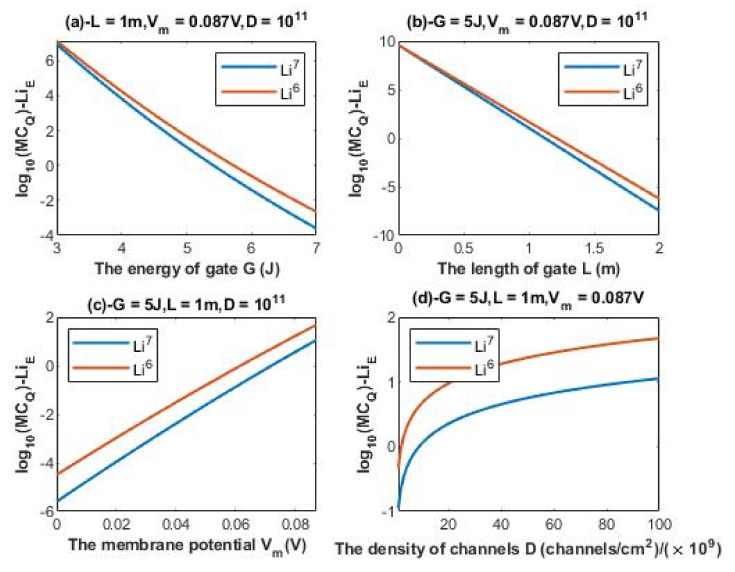
(**a**): The figure represents the relationship between the common logarithm of quantum membrane conductance of the extracellular lithium isotopes $\log_{10}(MC_{Q})-Li_{E}$ and the energy of the gate *G* by setting L=1 m, $V_{m}=0.087$ V and $D=10^{11}$ and across the range of *G* from 3 J to 7 J. (**b**): The figure represents the relationship between the common logarithm of quantum membrane conductance of the extracellular lithium isotopes $\log_{10}(MC_{Q})-Li_{E}$ and the length of the gate *L* by setting G=5 J, $V_{m}=0.087$ V and $D=10^{11}$ and across the range of *L* from 0 m to 2 m. (**c**): The figure represents the relationship between the common logarithm of quantum membrane conductance of the extracellular lithium isotopes $\log_{10}(MC_{Q})-Li_{E}$ and the membrane potential *V_m_* by setting G=5 J, L=1 m and $D=10^{11}$ and across the range of membrane potential from 0 V to 0.087 V. (**d**): The figure represents the relationship between the common logarithm of quantum membrane conductance of the extracellular lithium isotopes $\log_{10}(MC_{Q})-Li_{E}$ and the density of channels *D* by setting G=5 J, L=1 m and $V_{m}=0.087$ V and across the range of *D* from $10^{9}$ to $10^{11}$ channels/cm^2^.

According to Equations (20) and (21), the relationship between the common logarithm of quantum membrane conductance of extracellular lithium isotopes $\log_{10}(MC_{Q})-Li_{E}$ and the energy of the gate *G*, the length of the gate *L*, the membrane potential *V_m_*, and the density of channels *D* can be evaluated. See Figure 6.

The quantum membrane conductance of intracellular lithium isotopes:


(22) $$MC_{Q\left(Li-7\right)I}=3.88\times 10^{-2}e^{-19.2\frac{L}{G}\sqrt{{\left(G-0.64\right)}^{3}}}\times D$$

(23) $$MC_{Q\left(Li-6\right)I}=3.88\times 10^{-2}e^{-17.8\frac{L}{G}\sqrt{{\left(G-0.64\right)}^{3}}}\times D$$

The unit of quantum membrane conductance is mS/cm^2^.

**Figure 7:**
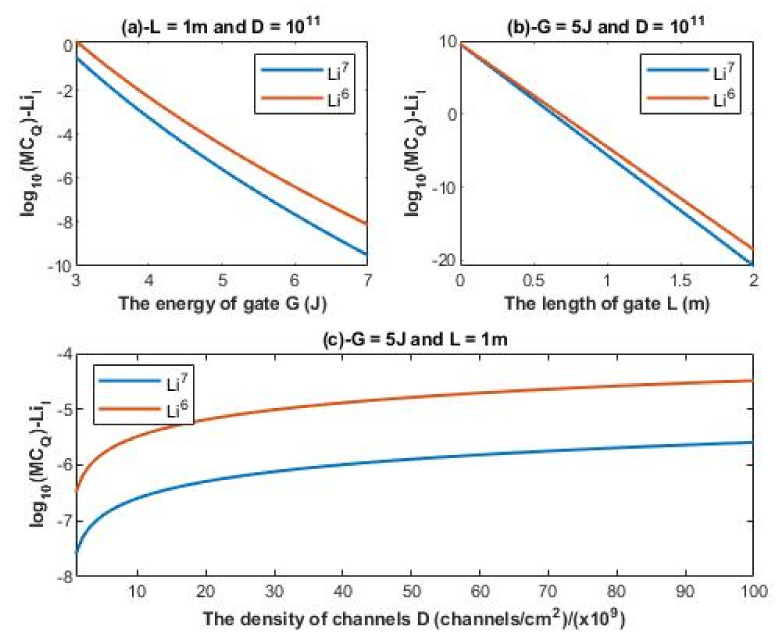
(**a**): The figure represents the relationship between the common logarithm of quantum membrane conductance of the intracellular lithium isotopes $\log_{10}(MC_{Q})-Li_{I}$ and the energy of the gate *G* by setting L=1 m and $D=10^{11}$ and across the range of *G* from 3 J to 7 J. (**b**): The figure represents the relationship between the common logarithm of quantum membrane conductance of the intracellular lithium isotopes $\log_{10}(MC_{Q})-Li_{I}$ and the length of the gate *L* by setting G=5 J and $D=10^{11}$ and across the range of *L* from 0 m to 2 m. (**c**): The figure represents the relationship between the common logarithm of quantum membrane conductance of the intracellular lithium isotopes $\log_{10}(MC_{Q})-Li_{I}$ and the density of channels *D* by setting G=5 J and L=1 m and across the range of *D* from $10^{9}$ to $10^{11}$ channels/cm^2^.

According to Equations (22) and (23), the relationship between the common logarithm of quantum membrane conductance of intracellular lithium isotopes $\log_{10}(MC_{Q})-Li_{I}$ and the energy of the gate *G*, the length of the gate *L*, and the density of channels *D* can be evaluated. See Figure 7.

### 3.4. The Influence of Classical Transport of Lithium Ions on the Resting Membrane Potential

To assess the influence of quantum tunneling of lithium isotopes on the resting membrane potential, the quantum version of GHK equation will be used. Additionally, the classical version of GHK equation will be used to compare the influence of classical transport to quantum transport of lithium ions on the resting membrane potential.

The resting membrane potential by considering the classical transport of lithium ions:

(24) $${\left[K\right]}_{E}MC_{K}+{\left[Na\right]}_{E}MC_{Na}+{\left[Li\right]}_{E}MC_{Li}=e^{-37.45V_{m}}(\left[K{]}_{I}MC_{K}+{\left[Na\right]}_{I}MC_{Na}+R{\left[Li\right]}_{E}MC_{Li}\right)$$

where *R* is the ratio of the intracellular concentration to the extracellular concentration of lithium ions. We will evaluate the influence of classical membrane conductance of lithium ions at the resting state (which is the same as the membrane conductance of sodium ions at the resting state) at different concentration ratios (R = 1–4) [38].

Considering $MC_{Na}=MC_{Li}=0.005$ mS/cm^2^ and the other variables of concentrations and potassium conductance are set to be the same as the reference values, Equation (24) becomes:


(25) $$2.71+{\left[Li\right]}_{E}0.005=e^{-37.45V_{m}}\left(70.07+R{\left[Li\right]}_{E}0.005\right)$$

Considering $MC_{Na}=MC_{Li}=0.05$ mS/cm^2^ and the other variables of concentrations and potassium conductance are set to be the same as the reference values, Equation (24) becomes:


(26) $$9.1+{\left[Li\right]}_{E}0.05=e^{-37.45V_{m}}\left(70.7+R{\left[Li\right]}_{E}0.05\right)$$

**Figure 8:**
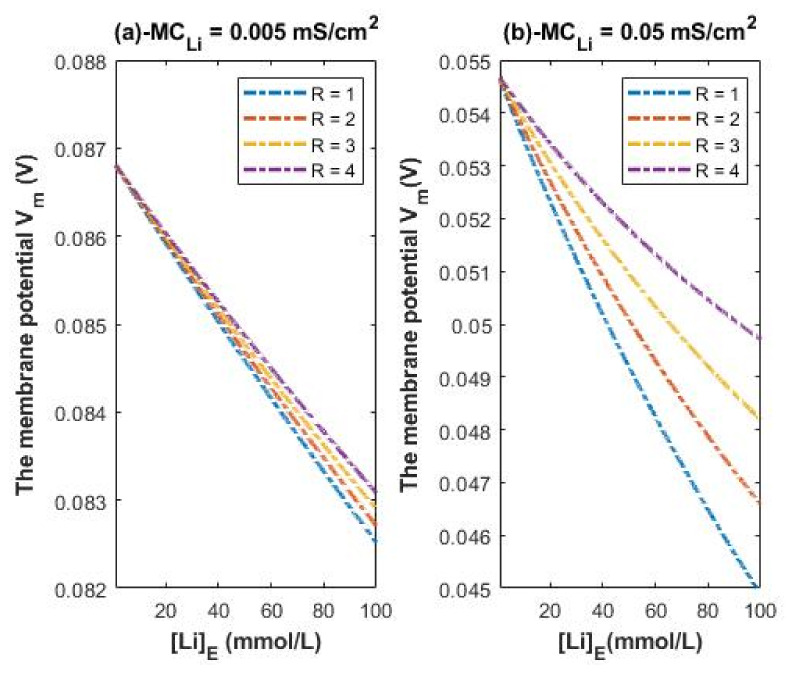
The figure represents the influence of classical transport of lithium ions on the resting membrane potential according to the concentration range 1–100 mmol/L. (**a**) The influence on resting membrane potential is evaluated when the membrane conductance of lithium is 0.005 mS/cm^2^ (the same as the conductance for sodium ions). (**b**) The influence on resting membrane potential is evaluated when the membrane conductance of lithium is 0.05 mS/cm^2^. This is applied on both lithium isotopes, assuming that the conductance ratio Li-6/Li-7 =1.006, which does not affect the mathematical graphs in this figure.

According to Equations (25) and (26), we evaluate the influence of a range of extracellular lithium concentration from 1–100 mmol/L on the resting membrane potential at different R values as presented graphically in Figure 8.

Figure 8 is applied on both lithium isotopes because the assumed conductance ratio between the two isotopes Li-6/Li-7 = 1.006 [23] (according to their diffusion) is too low to change the graphs in Figure 8 significantly. Hence, the classical transport of lithium ions does not differentiate between the two isotopes at this ratio.

Higher conductance ratio can be assumed according to the acceleration in the electric field across the channels using the following equation:$\;a=\frac{Eq}{m}$; where *a* is the acceleration, *E* is the electric field, *q* is the charge of lithium isotope, and *m* is the mass of isotope. All the variables in the equation are the same for the two isotopes except for the mass. The lower the mass, the higher the acceleration. Hence, the conductance ratio between Li-6/Li-7 can be assumed to be 1.17 (1.15/0.983).

**Figure 9:**
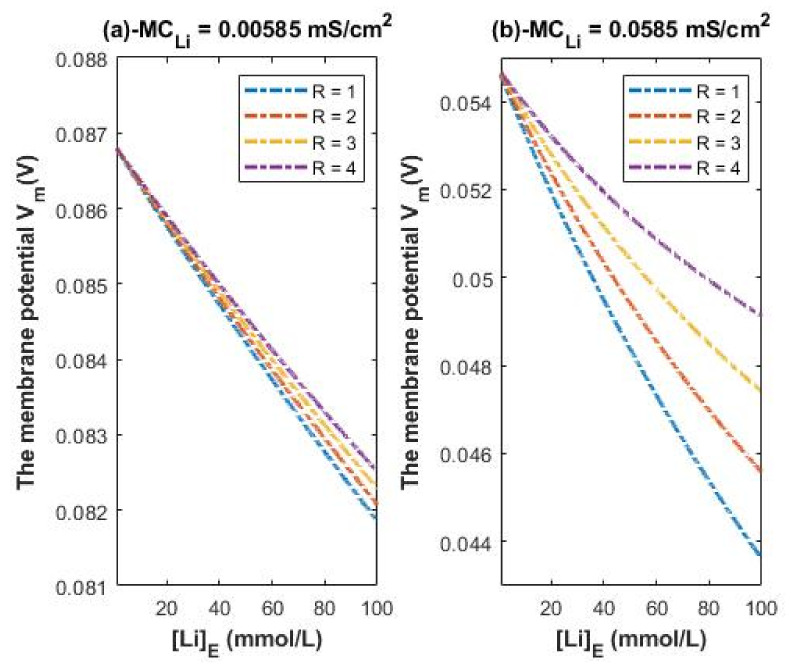
The figure represents the influence of classical transport of lithium ions on the resting membrane potential according to the concentration range 1–100 mmol/L for extracellular lithium ions. In this evaluation, the membrane conductance values in the previous figure (0.005 and 0.05) are increased by a factor of 1.17, which is the acceleration ratio between Li-6 and Li-7. (**a**) The influence on the resting membrane potential is evaluated when the membrane conductance of lithium ions Li-6 is 0.00585 mS/cm^2^, which is increased by 1.17 while the membrane conductance of sodium ions is still 0.005 mS/cm^2^. (**b**) The influence on the resting membrane potential is evaluated when the membrane conductance of lithium ions Li-6 is 0.0585 mS/cm^2^, which is increased by 1.17 while the membrane conductance of sodium ions is 0.05 mS/cm^2^.

Accordingly, the membrane conductance of Li-6 can be higher than 0.005 mS/cm^2^ and higher than 0.05 mS/cm^2^ by the factor 1.17. To represent the influence of such a factor on the membrane potential across the same range of extracellular lithium concentration 1–100 mmol/L, see Figure 9. The graphs in Figure 9 are plotted according to the Equations (25) and (26) except that we replace 0.005 by 0.00585 and 0.05 by 0.0585. Figure 9 represents the influence of the classical transport of Li-6 on the resting membrane potential. Thus, Figure 8 and Figure 9 can be compared to show the difference between the two isotopes in terms of the degree of depolarization from the perspective of classical transport. We take two conditions (at 0.005/0.00585 and at 0.05/0.0585) for the classical transport of lithium ions for the purposes of comparison with the quantum tunneling model of lithium ions as will be discussed later.

### 3.5. The Influence of Quantum Tunneling of Lithium Isotopes on the Resting Membrane Potential

On the other hand, the resting membrane potential under the influence of quantum tunneling of lithium ions Li-7:

(27) $$2.71+{\left[Li\right]}_{E}3.88\times 10^{-2}\times D\times e^{-19.2\frac{L}{G}\sqrt{{\left(G-16V_{m}-0.64\right)}^{3}}}=e^{-37.45V_{m}}\left(70.07+2{\left[Li\right]}_{E}3.88\times 10^{-2}\times D\times e^{-19.2\frac{L}{G}\sqrt{{\left(G-0.64\right)}^{3}}}\right)$$

The resting membrane potential under the influence of quantum tunneling of lithium ions Li-6:

(28) $$2.71+{\left[Li\right]}_{E}3.88\times 10^{-2}\times D\times e^{-17.8\frac{L}{G}\sqrt{{\left(G-16V_{m}-0.64\right)}^{3}}}=e^{-37.45V_{m}}\left(70.07+2{\left[Li\right]}_{E}3.88\times 10^{-2}\times D\times e^{-17.8\frac{L}{G}\sqrt{{\left(G-0.64\right)}^{3}}}\right)$$

The evaluation of the influence will be made by setting *G* = 5 J and *L* = 1 m over an extracellular lithium concentration range from 1–100 mmol/L at different values of channels density D. Additionally, the ratio between the intracellular and extracellular lithium ions is set to be 2 without considering other values because the values from 1 to 4 are indistinguishable to affect the membrane potential since the quantum tunneling and quantum conductance of the intracellular lithium ions are much lower than those for extracellular lithium ions as presented in the previous figures.

Accordingly, the resting membrane potential under the influence of quantum tunneling of Li-7 is determined by the following equation:

(29) $$2.71+{\left[Li\right]}_{E}3.88\times 10^{-2}\times D\times e^{-3.84\sqrt{{\left(4.36-16V_{m}\right)}^{3}}}=e^{-37.45V_{m}}\left(70.07+2{\left[Li\right]}_{E}3.88\times 10^{-2}\times D\times e^{-3.84\sqrt{{\left(4.36\right)}^{3}}}\right)$$

and the resting membrane potential under the influence of quantum tunneling of Li-6 is determined by the following equation:

(30) $$2.71+{\left[Li\right]}_{E}3.88\times 10^{-2}\times D\times e^{-3.56\sqrt{{\left(4.36-16V_{m}\right)}^{3}}}=e^{-37.45V_{m}}\left(70.07+2{\left[Li\right]}_{E}3.88\times 10^{-2}\times D\times e^{-3.56\sqrt{{\left(4.36\right)}^{3}}}\right)$$

**Figure 10:**
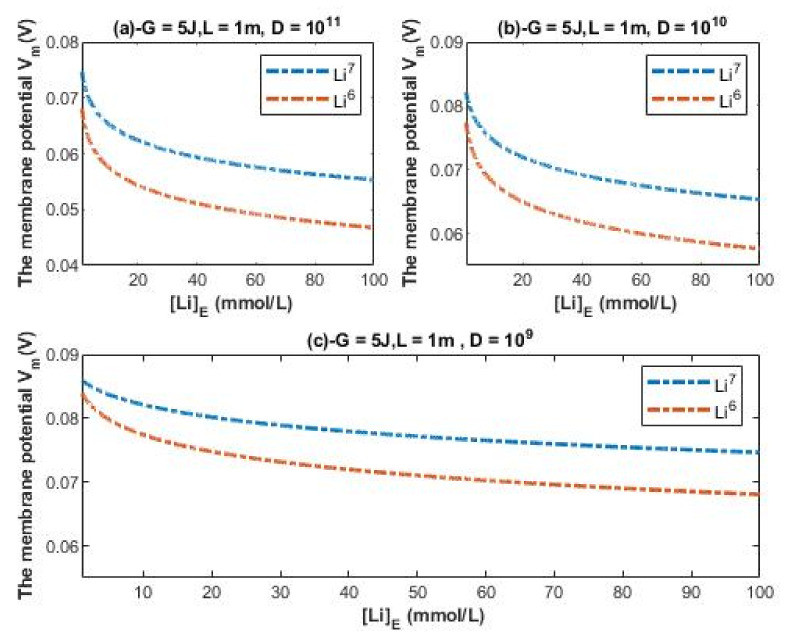
The figure represents the influence of quantum tunneling of lithium isotopes on the resting membrane potential according to the concentration range 1–100 mmol/L for extracellular lithium ions. The influence of lithium isotopes on the resting membrane potential is assessed under different values of sodium channels density *D* and by setting *G* = 5 J and *L* = 1 m. In the graph (**a**), $D=10^{11}$ channels/cm^2^, in the graph (**b**), $D=10^{10}$ channels/cm^2^, and in the graph (**c**), $D=10^{9}$ channels/cm^2^.

According to Equations (29) and (30), the relationship between the resting membrane potential under the influence of quantum tunneling of lithium isotopes and the extracellular lithium concentration can be evaluated as in Figure 10.

## 4. Discussion

The present study applies the model of quantum tunneling of lithium ions through the closed gate of voltage-gated sodium channels to understand the electrophysiological features of lithium ions, particularly the features related to the ability of lithium ions to depolarize the membranes. It evaluates the quantum tunneling probability and the quantum conductance of the two lithium isotopes under the influence of different factors including the energy of gate, the length of gate, the membrane potential, and the density of sodium channels.

Quantum tunneling is a property that enables ions to permeate through closed gates of channels and generate quantum currents. It occurs due to the wave nature of ions as predicted from quantum mechanics. These currents of ions have quantum conductance that reflects the quantum permeability of ions and determines the influence of quantum tunneling of ions on the resting membrane potential. In the context of quantum tunneling through closed channels, the probability of tunneling depends exponentially on the mass of ion, the energy of gate that blocks the permeation of ions, the length of gate, and the kinetic energy of the ion, which is determined by the voltage of the membrane and the temperature of the environment.

**Figure 11:**
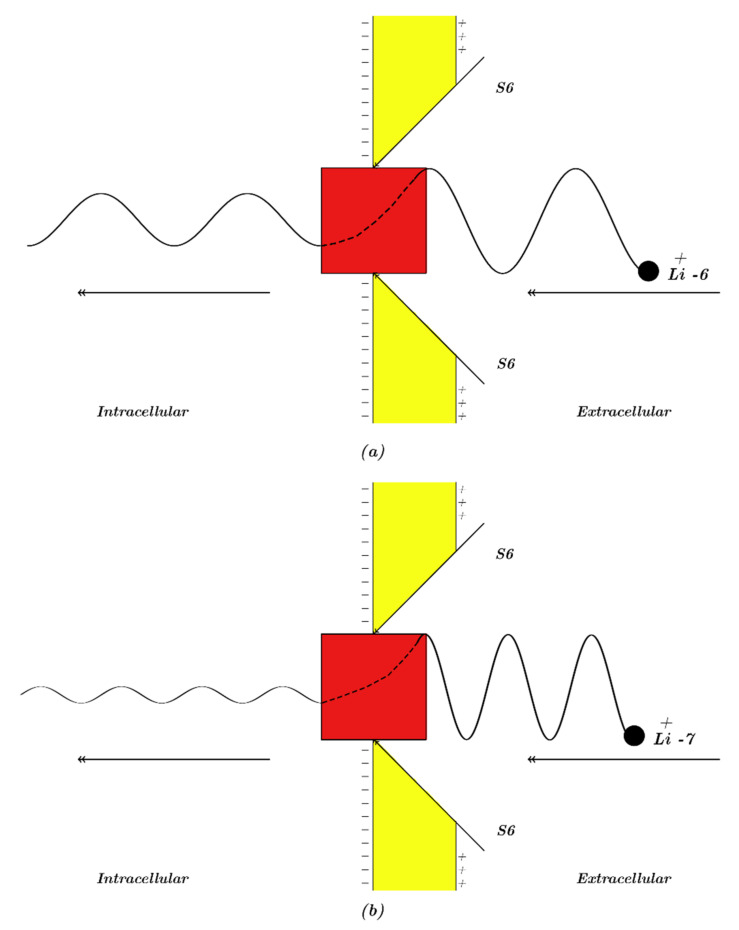
The figure represents a schematic diagram of the quantum tunneling of the extracellular lithium isotopes through the closed intracellular gate in which the dashed line represents the quantum tunneling process. (**a**): The quantum tunneling of the extracellular isotope Li-6. (**b**): The quantum tunneling of the extracellular isotope Li-7. The lithium isotope Li-6 has longer wave length and higher tunneling probability, which is represented by higher wave amplitude after passing the gate, while the lithium isotope Li-7 has shorter wave length and lower tunneling probability, which is represented by lower wave amplitude after passing the gate. The differences between the two isotopes in terms of wave length and wave amplitude after tunneling through the gate are attributed to the mass difference. In other words, the lower the mass, the longer the wave length and the higher the wave amplitude and vice versa.

It is clear from Figure 2 and Figure 3 that extracellular lithium ions have higher tunneling probability than the intracellular lithium ions. This is mainly attributed to the discrepancy of the kinetic energy between these two groups, as extracellular lithium ions obtain their kinetic energy by passing across the membrane voltage, which is negative inside compared to outside, and by thermal kinetic energy at body temperature, while intracellular lithium ions obtain the thermal kinetic energy only. The difference in tunneling probability between extracellular and intracellular lithium ions generates a ‘’quantum gradient’’ that favors the lithium to flow inside the cells. Interestingly, the isotopic effect of lithium ions on tunneling probability is evident as shown in Figure 2 and Figure 3. The tunneling probability of Li-6 ions is higher than the tunneling probability of Li-7 for both extracellular and intracellular lithium ions. The isotopic effect on the tunneling probability results from the mass difference between Li-6 and Li-7, hence, the isotope with the lower mass Li-6 has a higher tunneling probability than the isotope with the larger mass Li-7 because tunneling probability correlates inversely with the mass of ion. See Figure 11.

To demonstrate the differences between the intracellular and extracellular lithium ions and the difference between the two isotopes in terms of tunneling probability, a numerical description of Figure 2 and Figure 3 will be helpful.

### 4.1. The Numerical Description of the Quantum Tunneling Probability of Lithium Ions

#### 4.1.1. The Numerical Description of the Quantum Tunneling Probability of Extracellular Lithium Isotopes

A numerical description of the tunneling probability of extracellular lithium isotopes with respect to the energy of gate can be obtained from graph (a) of Figure 2. See Table 1. The graph (a) of Figure 2 shows that the difference in tunneling probability between the extracellular lithium isotopes increases as the value of *G* increases and vice versa.

A numerical description of the tunneling probability of extracellular lithium isotopes with respect to the length of gate can be obtained from graph (b) of Figure 2. See Table 2. The graph (b) of Figure 2 shows that the difference in tunneling probability between the extracellular lithium isotopes increases as the value of *L* increases and vice versa.

A numerical description of the tunneling probability of extracellular lithium isotopes with respect to the membrane potential can be obtained from graph (c) of Figure 2. The graph (c) of Figure 2 shows that the difference in tunneling probability between the extracellular isotopes is more maintained across the membrane potential range if it is compared with graphs (a) and (b) of Figure 2. See Table 3. 

#### 4.1.2. The Numerical Description of the Quantum Tunneling Probability of Intracellular Lithium Isotopes

A numerical description of the tunneling probability of intracellular lithium isotopes with respect to the energy of gate can be obtained from graph (a) of Figure 3. See Table 4. The graph (a) of Figure 3 shows that the difference in quantum tunneling probability between the intracellular lithium isotopes is more maintained if it is compared with the difference between the extracellular lithium isotopes in graph (a) of Figure 2.

A numerical description of the tunneling probability of intracellular lithium isotopes with respect to the length of gate can be obtained from graph (b) of Figure 3. See Table 5. The graph (b) of Figure 3 shows that the difference in quantum tunneling probability between the intracellular isotopes increases as the value of *L* increases and vice versa.

Interestingly, the quantum tunneling probability enables lithium ions to have a continuous spectrum of conductance values as exhibited in Figure 4 and Figure 5. On the other hand, the classical perspective of opening and closing the voltage-gated channels allows them to have two values of conductance: (1) zero conductance when they are closed; (2) certain value of conductance when the channel is open. Moreover, the quantum tunneling determines the quantum conductance of channels. Therefore, it is expected to observe differences between the intracellular and extracellular lithium ions and between the two lithium isotopes in terms of quantum conductance as they are observed in terms of tunneling probability. To demonstrate the continuous wide spectrum of conductance values and the differences between the intracellular and extracellular isotopes, a numerical description of Figure 4 and Figure 5 will be helpful.

### 4.2. The Numerical Description of the Quantum Conductance of Single Channel for Lithium Ions

#### 4.2.1. The Numerical Description of the Quantum Conductance of Single Channel for Extracellular Lithium Isotopes

A numerical description of the quantum conductance of single channel for extracellular lithium isotopes with respect to the energy of gate can be obtained from graph (a) of Figure 4. See Table 6.

A numerical description of the quantum conductance of single channel for extracellular lithium isotopes with respect to the length of gate can be obtained from graph (b) of Figure 4. See Table 7.

A numerical description of the quantum conductance of single channel for extracellular lithium isotopes with respect to the membrane potential can be obtained from graph (c) of Figure 4. See Table 8.

#### 4.2.2. The Numerical Description of the Quantum Conductance of Single Channel for Intracellular Lithium Isotopes

A numerical description of the quantum conductance of single channel for intracellular lithium isotopes with respect to the energy of gate can be obtained from graph (a) of Figure 5. See Table 9.

A numerical description of the quantum conductance of single channel for intracellular lithium isotope with respect to the length of gate can be obtained from graph (b) of Figure 5. See Table 10.

As the differences in quantum tunneling probability between the lithium isotopes were described previously, the differences in quantum conductance of single channel between the lithium isotopes can also be described. The difference in quantum conductance of single channel between the extracellular lithium isotopes increases as the values of *G* and *L* increase as in Figure 4a,b, while the difference between the extracellular isotopes is more maintained across the range of membrane potential as presented in Figure 4c. On the other hand, the difference in quantum conductance of single channel between the intracellular lithium isotopes, as in Figure 5a, is more maintained across the range of *G* if it is compared with that of extracellular lithium isotopes across the same range of *G*, while the difference in quantum conductance of single channel between the intracellular lithium isotopes increases as the value of *L* increases as in Figure 5b.

Obviously, the quantum conductance of single channel for extracellular lithium isotopes is higher than that for intracellular lithium isotopes. This is attributed to the discrepancy in the kinetic energy between extracellular and intracellular lithium ions. Additionally, the isotopic mass effect also influences the value of quantum conductance of single channel as it influences the values of tunneling probability. From the numerical description of the quantum conductance of single channel, it is clear that the quantum conductance takes a range of values and not just one value as expected from the classical understanding of voltage-gated channels [17]. From the classical measurements of single channel conductance for sodium channels, each type of sodium channel has a certain value of conductance that is within the order of magnitude $∼10^{-12}$ S [17]. Thus, the classical perspective of channels enables them to have a narrow range of conductance values, while the quantum perspective allows for a wide range of conductance values that might be lower or higher than the classical measurement and this is determined by factors such as the energy of the gate, the length of gate, the mass of the ion, and the kinetic energy of ions. The quantum model of channels shows wide variations in conductance of the same channel for different ions such as the quantum conductance of lithium ions and sodium ions through sodium channels, which are selective for them by the same degree. These variations between ions have been shown in the previous studies [26,27] and can be compared with the results presented here. Moreover, the quantum model shows considerable variations in conductance between the isotopes of the same ion as presented in this article. Classically, the single channel conductance for both lithium and sodium is expected to be $∼10^{-12}$ S [17] with almost no significant difference since the permeability ratio between them is around 1 and the difference between the two lithium isotopes is expected to be by the factor 1.006 up to 1.17, which is less than the difference expected by the quantum model as noticed from the figures in the results and the numerical description. Furthermore, the quantum model differentiates significantly between the extracellular and intracellular lithium ions in terms of single channel conductance as presented in the figures and the numerical description, while the classical perspective assigns the same certain value of conductance for both intracellular and extracellular lithium ions.

The eventual quantum property that comes from quantum tunneling is the quantum membrane conductance, which is determined by the quantum conductance of single channel and the number of channels available for tunneling. The quantum membrane conductance is an important factor to assess the ability of ions to affect the membrane potential of cells and to give reflection about the membrane permeability.

### 4.3. The Numerical Description of the Quantum Membrane Conductance of Lithium Ions

#### 4.3.1. The Numerical Description of the Quantum Membrane Conductance of Extracellular Lithium Isotopes

A numerical description of the quantum membrane conductance of extracellular lithium isotopes with respect to the energy of gate can be obtained from graph (a) of Figure 6. See Table 11.

A numerical description of the quantum membrane conductance of extracellular lithium isotopes with respect to the length of gate can be obtained from graph (b) of Figure 6. See Table 12.

A numerical description of the quantum membrane conductance of extracellular lithium isotopes with respect to the membrane potential can be obtained from graph (c) of Figure 6. See Table 13.

A numerical description of the quantum membrane conductance of extracellular lithium isotopes with respect to the density of channels can be obtained from graph (d) of Figure 6. See Table 14.

#### 4.3.2. The Numerical Description of the Quantum Membrane Conductance of Intracellular Lithium Isotopes

A numerical description of the quantum membrane conductance of intracellular lithium isotopes with respect to the energy of gate can be obtained from graph (a) of Figure 7. See Table 15.

A numerical description of the quantum membrane conductance of intracellular lithium isotopes with respect to the length of gate can be obtained from graph (b) of Figure 7. See Table 16.

A numerical description of the quantum membrane conductance of intracellular lithium isotopes with respect to the density of channels can be obtained from graph (c) of Figure 7. See Table 17.

The observations that are made on quantum tunneling probability and quantum conductance of single channel are also applied on the quantum membrane conductance. The difference in quantum membrane conductance between the extracellular lithium isotopes increases as the values of *G* and *L* increase as presented in graph (a) and graph (b) of Figure 6, while the difference between the extracellular isotopes is more maintained across the range of membrane potential and the range of channels density *D* as presented in graph (c) and graph (d) of Figure 6. On the other hand, the difference in quantum membrane conductance between the intracellular lithium isotopes, as in graph (a) and graph (c) of Figure 7, is more maintained across the range of *G* and the range of channels density *D*, while the difference in quantum membrane conductance between the intracellular lithium isotopes increases as the value of *L* increases as presented in graph (b) of Figure 7. Moreover, the quantum membrane conductance of extracellular lithium isotopes is higher than the quantum membrane conductance of intracellular lithium ions, whereas the classical membrane conductance is the same for intracellular and extracellular ions.

The quantum model is more dynamic when compared to the classical model. In the present study, the main style of investigation is studying the influence of a certain factor and putting the other factors as constants as stated before. Consequently, in each figure the results of quantum properties can be estimated and further elaborated rationally to be increased or decreased as the setting values of factors change. As we said before, the quantum model allows a wide range of values to be used for the quantum tunneling and the quantum conductance.

According to our reference values of the leak membrane conductance of sodium and potassium ions at the resting state, the quantum membrane conductance of lithium ions (specifically extracellular lithium ions), as evident in the figures and in the numerical descriptions, can be much higher than the classical leak conductance of sodium ions (0.005 mS/cm^2^) and even the conductance of potassium ions (0.5 mS/cm^2^). This can explain the high resting permeability of lithium ions [1,2], which could not be explained by classical electrophysiology. Furthermore, the significant discrepancy in quantum conductance between the intracellular and extracellular lithium ions will result in significant flow of extracellular lithium ions to inside the cells. This inward flow of lithium ions tends to depolarize the resting membrane potential.

Eventually, by obtaining the values of quantum membrane conductance of lithium ions, the influence of quantum tunneling of lithium ions on the resting membrane potential can be assessed. First, the influence of classical transport of lithium ions through open channels (leak conductance) will be considered. Figure 8 shows the influence of the classical leak conductance of lithium ions on the membrane potential at the resting state at two conditions: (a) $MC_{Li}=0.005$ mS/cm^2^ (the conductance of sodium is also 0.005 mS/cm^2^ and the conductance of potassium is 0.5 mS/cm^2^); and (b) $MC_{Li}=0.05$ mS/cm^2^ (the conductance of sodium is also 0.05 mS/cm^2^ and the conductance of potassium is 0.5 mS/cm^2^). We provide two conditions to show the degree of depolarization at different conductance values.

In this section, the degree of depolarization will be calculated to assess the extent of depolarization when adding lithium ions. The degree of depolarization can be calculated by the following equation:

(31) $$DD=V_{m}\left(initial\right)-V_{m}\left(Li\right)$$

where *DD* is the degree of depolarization,$\;V_{m}\left(initial\right)$ is the membrane potential before adding lithium ions, and $V_{m}\left(Li\right)$ is the membrane potential after adding lithium at certain lithium concentration.

### 4.4. The Numerical Description of the Depolarization Induced by the Classical Transport of Lithium Ions

A numerical description of the membrane potential values with respect to the extracellular lithium concentration can be obtained based on the classical transport of lithium ions as in graph (a) of Figure 8. See Table 18.

Based on Table 18, a numerical description of the depolarization degrees can be obtained. See Table 19.

A numerical description of the membrane potential values with respect to the extracellular lithium concentration can be obtained based on the classical transport of lithium ions as in graph (b) of Figure 8. See Table 20.

Based on Table 20, a numerical description of the depolarization degrees can be obtained. See Table 21.

The permeability ratio between the two isotopes can be estimated to be 1.006 according to their diffusion [23] and this ratio is not high enough to change the leak conductance for the two isotopes and thus is not enough to show change in the effect on the resting membrane potential. Therefore, Figure 8 is valid for both isotopes with no significant difference between them in the degree of depolarization. However, if we take the ratio to be 1.17 (the acceleration ratio Li-6/Li-7 in regular electric field according to the equation a = qE/m), Figure 9 can show a slight difference between the two isotopes if it is compared with Figure 8 Hence, Figure 9 represents the influence of Li-6 on the resting membrane potential by considering the ratio factor 1.17 and it can be compared with Figure 8 to show the slight difference between the two isotopes.

A numerical description of the membrane potential values with respect to the extracellular lithium concentration can be obtained based on the classical transport of lithium ions Li-6 as in graph (a) of Figure 9. See Table 22.

Based on Table 22, a numerical description of the depolarization degree can be obtained. See Table 23.

A numerical description of the membrane potential values with respect to the extracellular lithium concentration can be obtained based on the classical transport of lithium ions Li-6 as in graph (b) of Figure 9. See Table 24.

Based on Table 24, a numerical description of the depolarization degrees can be obtained. See Table 25.

Based on the previous numerical descriptions, the classical perspective of lithium permeability cannot strongly differentiate between the two isotopes because the difference in the degree of depolarization is small or even absent. Moreover, it has been shown that lithium ions can depolarize the resting membrane potential from around −75 mV to around −49 mV [1], which represents depolarization by 26 mV. Moreover, it can depolarize the membrane potential from −85 mV to −59 mV [1], which also represents depolarization by 26 mV. These depolarizations are induced at extracellular lithium concentration of 100 mmol/L and intracellular lithium concentration of 120 mmol/L [1]. As a result, classical electrophysiology cannot explain the experimental observation of the large depolarization induced by lithium, which is about 26 mV at extracellular lithium concentration of 100 mmol/L, because the classical calculations in the present study show that the range of depolarization by Li-7 and Li-6 can range from 4 mV to 10 mV and 5 mV to 11 mV, respectively. Additionally, the therapeutic concentration of lithium in the clinical practice is about 1 mmol/L. The classical calculations show that lithium at this low extracellular concentration fails to affect the membrane potential and induce membrane depolarization. This is said because it is assumed that lithium exhibits its therapeutic effect via membrane depolarization [3,5]. So, the presence of a certain mechanism that predicts membrane depolarization at the low extracellular lithium concentration of 1 mmol/L is vital to support the causal link between membrane depolarization and the effects of lithium. This causal link will be further supported in the upcoming sections and it will not be restricted to the effects at low therapeutic concentrations, but it will include the effects at higher concentrations investigated experimentally.

The quantum model of lithium ions can provide a reasonable explanation for the large depolarization induced by lithium at high concentration and an explanation for the depolarization induced at the low therapeutic concentration. Moreover, it can account significantly for the differences between the two isotopes if Figure 8, Figure 9 and Figure 10 are compared.

### 4.5. The Numerical Description of the Depolarization Induced by the Quantum Tunneling of Lithium Ions

A numerical description of the membrane potential values with respect to the extracellular lithium concentration can be obtained based on the quantum transport of lithium ions as in graph (a) of Figure 10. See Table 26.

Based on Table 26, a numerical description of the depolarization degrees can be obtained. See Table 27.

A numerical description of the membrane potential values with respect to the extracellular lithium concentration can be obtained based on the quantum transport of lithium ions as in graph (b) of Figure 10. See Table 28.

Based on Table 28, a numerical description of the depolarization degrees can be obtained. See Table 29.

A numerical description of the membrane potential values with respect to the extracellular lithium concentration can be obtained based on the quantum transport of lithium ions as in graph (c) of Figure 10. See Table 30.

Based on Table 30, a numerical description of the depolarization degrees can be obtained. See Table 31.

The graphs of Figure 10 are plotted assuming that the concentration ratio between the intracellular and extracellular lithium ions is 2 without considering other values because the quantum membrane conductance of intracellular lithium ions is much lower than that for extracellular lithium ions, making the graphs almost identical by substituting any value from 1 to 4. This makes the quantum model distinguishable from the classical model, which is more affected by the concentration ratio as shown before. Notably, the effect on the resting membrane potential by quantum tunneling is more dynamic and depends on many factors. Hence, in each graph of Figure 10, the values of membrane potential can be rationally estimated to be less or more by changing the setting values in each figure, or simply applying the equations to obtain the exact value.

Based on the previous numerical descriptions, quantum tunneling model of lithium ions can explain the large depolarization induced by lithium at high concentrations and can explain the depolarization induced at low therapeutic concentrations. Moreover, the quantum model differentiates more significantly between the two lithium isotopes in terms of the degree of depolarization if it is compared with the classical model. This may predict that lithium isotopes differ from each other in terms of the effectiveness of the cellular effects because it is assumed here in the present study that lithium mediates its actions via membrane depolarization.

Interestingly, it was found that Li-6 is more effective in reducing ketamine-induced hyperactivity when compared to Li-7 [39]. Our study gives consistent results as it shows that Li-6 can depolarize the resting membrane potential more significantly than Li-7. The higher depolarization induced by Li-6 is assumed to be the cause behind the higher efficacy of Li-6 in reducing hyperactivity in the animal model and maybe in bipolar patients. In addition to that, the quantum model can explain the dissimilar biochemical and behavioral effects [40]. The cellular uptake and the intracellular accumulation of lithium ions are higher for Li-6 [40], which can be explained by the higher quantum tunneling of Li-6 into the cells. Moreover, Li-6 was shown to produce a stronger effect in reducing motility and a stronger effect on mental behavior [40] and this may be attributed to the larger depolarization induced by Li-6 due to its higher quantum conductance when compared to Li-7.

In the following sections, we are going to focus on several cellular effects mediated and influenced by both lithium ions and membrane potential. The major aim of these sections is to shed light on the consistency between these cellular effects and support the hypothesis of the study. The present study represents the first attempt to unify the effects of lithium under the influence of one action, which is membrane depolarization. Therefore, further studies are required to test such unification and address the factors that determine the strength of such associations.

### 4.6. Cellular Growth and Cancer Cells Proliferation

Lithium has proliferative and anti-proliferative effects on cells depending on the cell type and whether the cell is normal or cancerous [9]. This also applies to depolarization because the proliferation of certain cell types is stimulated by depolarization, but the proliferation of other cell types is inhibited by depolarization [16].

Interestingly, the differential effect of membrane depolarization on cellular proliferation can be attributed to the presence of electrical checkpoints of the cell cycle that control the DNA synthesis and mitosis. Moreover, these checkpoints are governed by changes in the membrane potential which fluctuate between hyperpolarization and depolarization during the cell cycle. Additionally, the exact value of membrane potential threshold of depolarization and hyperpolarization varies according to the cell type [41,42]. Several examples can be listed to show the consistency between the effects of membrane depolarization and the effects of lithium ions on cellular growth and cancer cells proliferation. Membrane depolarization is either stated clearly as in the original reference or is indirectly concluded by the blockage of potassium channels [17,18,42].

1. Lithium and membrane depolarization induce pulmonary artery smooth muscle hypertrophy via inhibition of Glycogen Synthase Kinase-3-beta (GSK-3*β*) [43,44,45,46].
2. It was shown that decreasing the activity of voltage-gated potassium channels results in membrane depolarization and inhibition of proliferation for RPMI-8226 multiple myeloma cell line [47]. On the other hand, lithium chloride inhibits the same cell line RPMI-8226 survival and triggers apoptosis in multiple myeloma via activation of the wnt/β-catenin pathway in a dose-dependent manner [48]. Interestingly, potassium channels restrict the wnt/β-catenin pathway activity and the inhibition of potassium channels, which results in membrane depolarization, potentiates the activity of the wnt/β-catenin pathway [49]. Here is a consistent correlation that indicates lithium inhibits the proliferation of RPMI-8226 cell line via membrane depolarization that potentiates the activity of the wnt/β-catenin pathway.
3. The depolarization of cellular membrane of neuroblastoma cells SH-SY5Y activates cyclin-dependent kinase 2 (cdk2) which phosphorylates retinoblastoma protein. Phosphorylated retinoblastoma proteins promote the progression of cell cycle and therefore cell proliferation [50]. On the other hand, lithium acetoacetate or lithium chloride have induced cellular proliferation in the neuroblastoma cell line SH-SY5Y, which is the same line stimulated by membrane depolarization [9].
4. Membrane depolarization was shown to enhance the apoptosis of human melanoma cells induced by tumor-necrosis-factor-related apoptosis-inducing ligand (TRAIL), and the drugs that result in membrane depolarization such as K_ATP_ channel inhibitors play a role in the tumor-selective cytotoxicity [51]. On the other hand, lithium has an anti-proliferative effect against melanoma cells [52,53]. Consistently, GSK-3*β* inhibitors such as lithium chloride enhance TRAIL-mediated apoptosis in human gastric adenocarcinoma and human prostate cancer cell lines [54,55]. Even though the enhancement of TRAIL-mediated apoptosis by lithium is not tested on melanoma cells, the consistent correlations predict such an enhancement on melanoma cells. Here is another convincing observation that lithium can enhance apoptosis via membrane depolarization, which facilitates TRAIL-induced cell death.
5. The depolarization of cell membrane by high extracellular potassium concentration or blocking the voltage-gated potassium channels [56] stimulates the proliferation of breast cancer cells MCF-7 (hormone-dependent) and MDA-MB-123 (hormone-independent) [57]. On the other hand, lithium chloride stimulated the proliferation of the estrogen-dependent MCF-7 breast cancer cell line by lithium concentration up to only 5 mM, but these concentrations did not stimulate the proliferation of MDA-MB-123, which is hormone-independent [58]. It was possible that if lithium concentration was increased more than 5 mM, the stimulation effect would involve the hormone-independent cell lines since larger depolarization will be induced.
6. Blockage of calcium-activated potassium channels inhibits endometrial cancer cells proliferation [59,60]. Blocking the actions of GSK-3*β* by lithium would result in inhibition of tumor growth [61].
7. The proliferation of hepatocellular carcinoma (HCC) cell line Hep G2 is inhibited by blocking the intermediate conductance calcium-activated potassium channel Kca3.1 using TRAM-34. TRAM-34 also inhibited its migration and promoted its apoptosis [62]. On the other hand, lithium induced cell cycle arrest and apoptosis in HCC-29 cells [63].

It is difficult to cover all the studies that investigated the proliferative effects of lithium and membrane depolarization in this article because this goes beyond the aim of the present study. In the upcoming sections, we continue to show the similarity in different cellular effects.

### 4.7. Stem Cells Differentiation

Since most cells differentiate via hyperpolarization [16], we expect that lithium inhibits differentiation since it induces membrane depolarization.

1. It was shown that hyperpolarization, which is made by overexpression of potassium channels, can induce the differentiation of human cardiomyocyte progenitor cells (CMPCs) into spontaneously beating cardiomyocytes [64]. On the other hand, lithium chloride inhibited the differentiation of mouse embryonal stem (ES) cells into cardiac cells [65].
2. It was suggested that depolarization inhibited osteogenic differentiation [66,67,68]. On the other hand, lithium ions also inhibited osteogenic differentiation [69,70].

### 4.8. Neuroprotection

Neuroprotection is well known to be mediated by lithium ions, especially in treating bipolar patients [71]. Additionally, lithium is a candidate drug for neurodegenerative diseases such as: Alzheimer’s disease (AD), amyotrophic lateral sclerosis (ALS), and Parkinson’s disease (PD) [71]. The major molecular target of lithium to exhibit its neuroprotective effects is the inhibition of GSK-3*β* [72]. The inhibition of GSK-3*β*, as we said before, can be mediated by membrane depolarization, supporting its relation to the neuroprotective effects of lithium.

It has been shown that resting membrane potential of leukocytes in bipolar patients is hyperpolarized [73]. This supports the hypothesis of therapeutic depolarization mediated by lithium ions, particularly in bipolar patients [3,5]. Moreover, membrane depolarization of arrested neurons leads to making them mitotically active [74] and promotes the differentiation of midbrain dopamine neurons from neural precursor cells [75]. Consistently, lithium enhances the proliferation of neural stem cells and their differentiation into dopaminergic neurons [76]. This effect of lithium is thought to be mediated by the activation of wnt/β-catenin pathway, which can be stimulated by membrane depolarization as was discussed before. Furthermore, membrane depolarization increases the dopamine content prior to its release by the process of vesicular hyper-acidification [77]. Interestingly, lithium was able to prevent N-methyl-4-phenyl-1,2,3,6-tetrahydropyridine (MPTP)-induced depletion of striatal dopamine (DA) [78]. The beneficial effects of lithium and membrane depolarization on the dopaminergic neurons are assumed to be responsible for the neural regeneration in Parkinson’s disease.

### 4.9. Wound Healing

Lithium chloride activates wnt/β-catenin which mediates wound healing by deposition of collagen, hair follicle formation, and re-epithelization [79]. Additionally, lithium combined with negative pressure accelerates wound healing by activation of the wnt/β-catenin pathway [11]. Moreover, lithium upregulated wound healing genes and facilitated various stages of wound healing [80]. On the other hand, several studies showed that membrane depolarization plays an important role in wound healing [16,81,82].

### 4.10. Immunomodulation

The hypothesis that states that lithium modulates the functions of the immune system via membrane depolarization has been addressed before, particularly in the context of COVID-19 [83].

Here we demonstrate a strong correlation which indicates that lithium modulates the activity of macrophages via membrane depolarization. It has been shown that membrane depolarization shifts the phenotype of macrophages from the pro-inflammatory phenotype (M1) to the anti-inflammatory and wound-healing phenotype (M2). This was evident by an increase in IL-10 and wound healing marker Arg-1 and a decrease in the levels of pro-inflammatory markers such as TNF-alpha and IL-1beta [84]. Consistently, lithium, which is assumed to induce membrane depolarization, was able to upregulate the immunomodulatory and wound-healing genes (M2) of macrophages such as IL-10 and Arg-1 and downregulated the pro-inflammatory genes (M1) such as IL-1beta [85].

The membrane potential of T and B lymphocytes fluctuates between depolarization and hyperpolarization during the mitogenic stimulation. This process is initiated by membrane depolarization [86]. On the other hand, lithium promotes the production of immunoglobulins IgG and IgM produced by B-lymphocytes [87]. Moreover, lithium augments the activity of T- lymphocytes in response to mitogens and antigens; however, lithium can suppress the production of T lymphocytes by induction thymus involution [88]. The dual effect of lithium on T-lymphocytes is reasonable since both depolarization and hyperpolarization are required for T cells function, as we said before. Consistently, the inhibitory effect of lithium on T cells, which is assumed to be mediated by membrane depolarization, is also found to be the result of potassium channels blockers, which lead to membrane depolarization [89].

Even though we mentioned many examples of the cellular effects that support strong consistency between the effects of lithium and membrane depolarization, further studies are required to give more extensive details on the extent and the time course of depolarization and to include other common cellular effects between lithium and depolarization. It is impossible to include all such details in one study, especially since the present study includes another major aspect, which is the quantum tunneling-induced membrane depolarization. Therefore, we aimed, by mentioning these examples, to hypothesize a novel link between membrane depolarization and lithium and to attract the attention of the researchers in this field to this hypothesis so that further studies can be conducted to test the scientific validity of the hypothesis.

To make a bridge between our theoretical approach and any future experimental studies, we propose some experimental approaches that can be used to link the quantum tunneling model with electrophysiological measurements:

1. The conductance and electrical currents through closed state of voltage-gated channels have been documented before in the literature [90,91]. These electrophysiological observations are more consistent with the mechanism of quantum tunneling as this mechanism is based on the ability of ions to tunnel through closed state of channels. Accordingly, if the strategy of inducing mutations in the residues of hydrophobic gate of channels is adopted [91], this will offer a great opportunity to link the quantum tunneling model with the electrophysiological measurements. Inducing mutations should be performed to lower the energy of the gate *G* and maintain the channels in the closed state [91]. When the energy of gate *G* decreases, the quantum tunneling of lithium ions will increase as predicted from the model of quantum tunneling. So, these mutated closed channels will offer valuable experimental data to support the mechanism of quantum tunneling. If the mechanism of quantum tunneling is scientifically valid, the measurements of single channel conductance values will be consistent with the calculated values in the present paper. In other words, different mutated closed channels will have different values of single channel conductance according to the mutation and its influence on the energy of gate. Interestingly, these quantum values of single channel conductance can be lower or even higher than the value of single channel conductance when the channel is open. If such observations are made, they will provide reasonable evidence on quantum tunneling through closed channels. For example, if the electrophysiological measurements indicate that the conductance value of the open state of sodium channel is $1\times 10^{-12}$ S, then the measurements of conductance of closed mutated channels may show conductance values higher than $1\times 10^{-12}$ S and this supports the existence of quantum tunneling as the drop in the energy of gate by mutations enhances quantum tunneling. Moreover, the quantum tunneling model can assign conductance values for the closed mutated channels unlike the classical model which assigns zero conductance values for the closed channels. Thus, if the measurements indicate that closed mutated channels can have non-zero conductance values, this will give consistent evidence for the quantum tunneling model.
  In this reference [90], the values of conductance for the closed potassium channel range between 0 S and $1.6\times 10^{-16}$ S, but this range cannot be compared with the ranges of our study because our calculations are applied on lithium ions not potassium ions. This is said because the equations of tunneling probability differentiate significantly between ions in terms of mass. For example, if the mass of potassium ion $m_{K}=6.5\times 10^{-26}$ Kg, G = 5 J, L = 1 m, and V_m_= 0.087 V are substituted in Equation (8), the quantum conductance for potassium ions will be $2.13\times 10^{-25}$ S, which is lower than the values of lithium ions presented in Table 6, but it is within the range documented in this reference [90].
2. As the quantum model differentiates more significantly between the two lithium isotopes, it is expected that the electrophysiological measurements will yield two different values of single channel conductance for the two lithium isotopes Li-7 and Li-6. This is a unique quality of quantum tunneling that can be used to give evidence on the existence of quantum tunneling of lithium ions. To provide a concrete approach for this unique quality of quantum tunneling, the ratio between the two isotopes Li-6/Li-7 can be calculated.

The ratio between the extracellular isotopes Li-6/Li-7 (*E*) in terms of tunneling probability and quantum conductance can be calculated as the following:

(32) $$\frac{Li-6}{Li-7}\left(E\right)=\frac{T_{Q\left(Li-6\right)E}}{T_{Q\left(Li-7\right)E}}=\frac{C_{Q\left(Li-6\right)E}}{C_{Q\left(Li-7\right)E}}=\frac{MC_{Q\left(Li-6\right)E}}{MC_{Q\left(Li-7\right)E}}$$

Then:

(33) $$\frac{Li-6}{Li-7}\left(E\right)=e^{1.4\times \frac{L}{G}\sqrt{{\left(G-16V_{m}-0.64\right)}^{3}}}$$

On the other hand, the ratio between the intracellular isotopes Li-6/Li-7 (*I*) in terms of tunneling probability and quantum conductance can be calculated by the following equation:

(34) $$\frac{Li-6}{Li-7}\left(I\right)=\frac{T_{Q\left(Li-6\right)I}}{T_{Q\left(Li-7\right)I}}=\frac{C_{Q\left(Li-6\right)I}}{C_{Q\left(Li-7\right)I}}=\frac{MC_{Q\left(Li-6\right)I}}{MC_{Q\left(Li-7\right)I}}$$

Then:

(35) $$\frac{Li-6}{Li-7}\left(I\right)=e^{1.4\times \frac{L}{G}\sqrt{{\left(G-0.64\right)}^{3}}}$$

**Figure 12:**
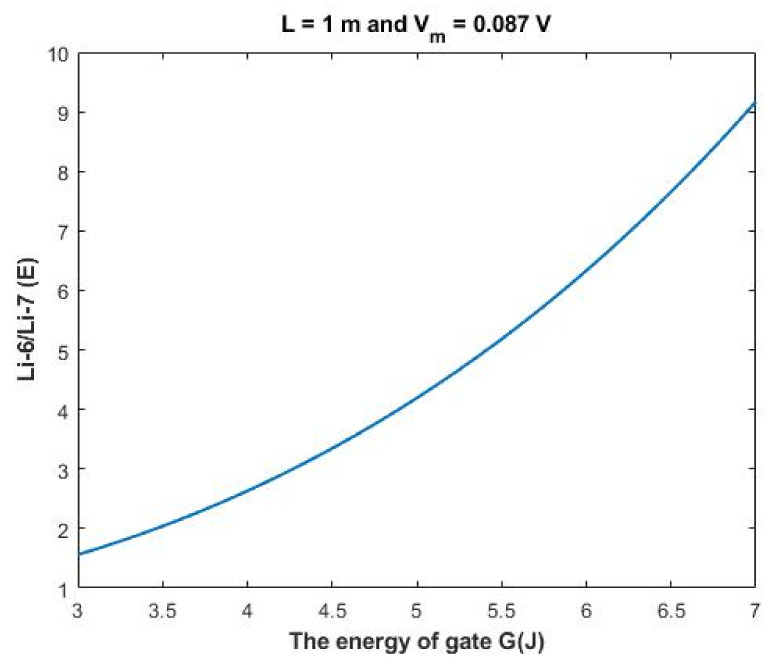
The figure represents the relationship between the ratio of the extracellular lithium isotopes Li-6/Li-7 (*E*) and the energy of gate *G* when setting L=1 m and $V_{m}=0.087$ V.

Based on Figure 12, a numerical description of the relationship can be obtained. See Table 32.

The rate of change in the ratio with respect to the energy of gate *A (G)* can be calculated by the following equation:

(36) $$A\left(G\right)=\frac{\frac{Li-6}{Li-7}\left(G=7\right)-\frac{Li-6}{Li-7}\left(G=3\right)}{4}$$

**Figure 13:**
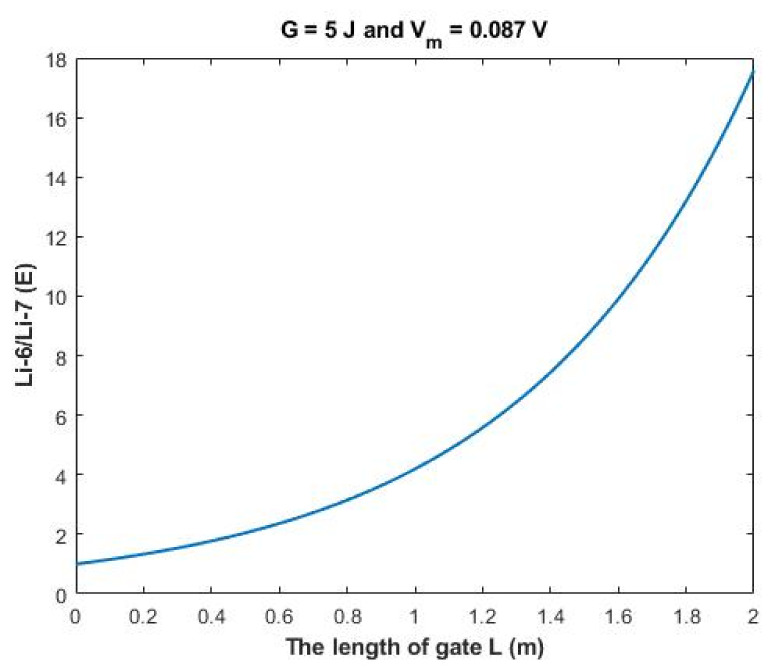
The figure represents the relationship between the ratio of the extracellular lithium isotopes Li-6/Li-7 (*E*) and the length of gate *L* when setting G=5 J and $V_{m}=0.087$ V.

Based on Figure 13, a numerical description of the relationship can be obtained. See Table 33.

The rate of change in the ratio with respect to the length of gate *A (L)* can be calculated by the following equation:

(37) $$A\left(L\right)=\frac{\frac{Li-6}{Li-7}\left(L=2\right)-\frac{Li-6}{Li-7}\left(L=0\right)}{2}$$

**Figure 14:**
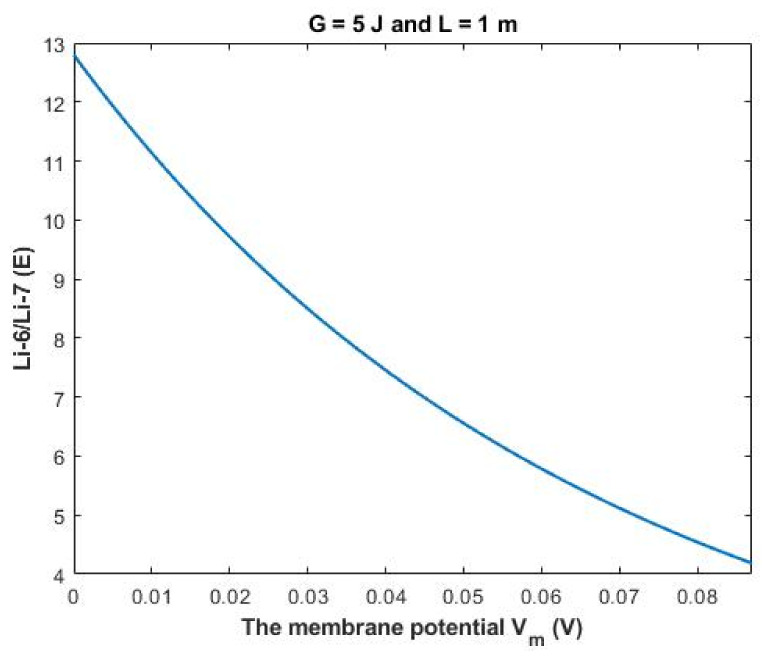
The figure represents the relationship between the ratio of the extracellular lithium isotopes Li-6/Li-7 (*E*) and the membrane potential when setting G=5 J and L=1 m.

Based on Figure 14, a numerical description of the relationship can be obtained. See Table 34.

The rate of change in the ratio with respect to the membrane potential *A (V_m_)* can be calculated by the following equation:

(38) $$A\left(V_{m}\right)=\frac{\frac{Li-6}{Li-7}\left(V_{m}=87\;\mathrm{mV}\right)-\frac{Li-6}{Li-7}\left(L=0\;\mathrm{mV}\right)}{87}$$

**Figure 15:**
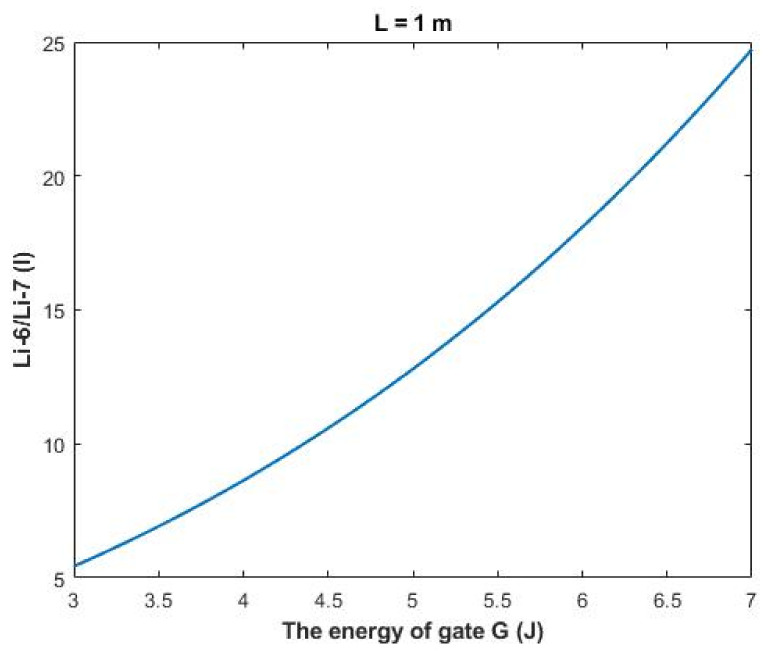
The figure represents the relationship between the ratio of the intracellular lithium isotopes Li-6/Li-7 (I) and the energy of gate *G* when setting L=1 m.

Based on Figure 15, a numerical description of the relationship can be obtained. See Table 35.

**Figure 16:**
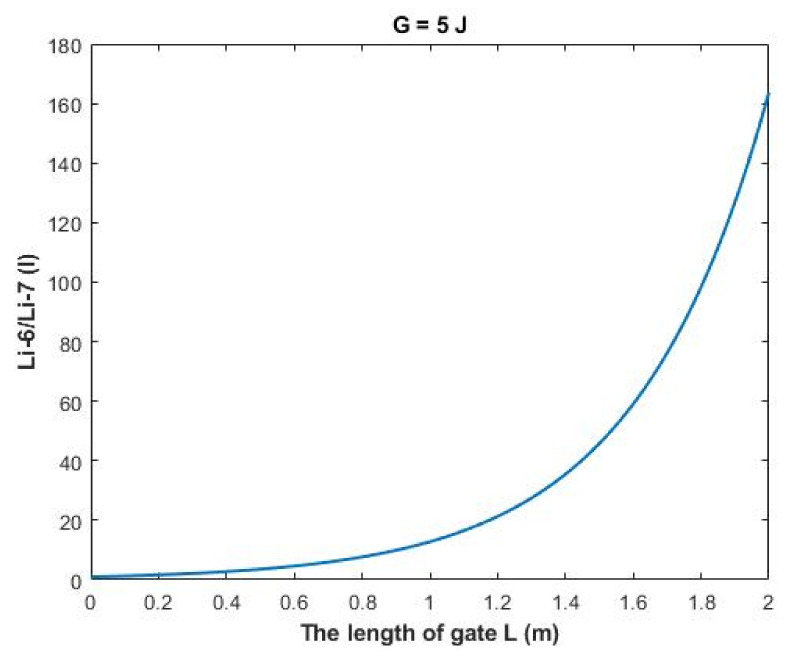
The figure represents the relationship between the ratio of the intracellular lithium isotopes Li-6/Li-7 (*I*) and the length of gate *G* when setting G=5 J.

Based on Figure 16, a numerical description of the relationship can be obtained. See Table 36.

Based on the previous figures and numerical descriptions, the following approaches can be used to test the quantum tunneling model of lithium ions:

- The quantum tunneling model predicts different isotope ratios for extracellular and intracellular lithium ions, while the classical model assigns the same ratio for the extracellular and intracellular lithium isotopes.
- The quantum tunneling model can assign much higher values of isotope ratio if it is compared with the classical model which predicts the ratio to be limited to the range 1.006–1.17. This explains the ability of the quantum tunneling model to differentiate between the two isotopes.
- The quantum tunneling model also predicts the rates at which the isotope ratio changes with respect to the energy of the gate *G*, the length of the gate *L*, and the membrane potential *V_m_*.

As every study has certain limitations that should be mentioned, our mathematical model and equations do not include the temporal aspect of quantum tunneling and its related depolarization since the equations used in this study do not include the time factor. Therefore, in this study we aimed to investigate the extent and degree of depolarization induced by the quantum tunneling of lithium ions according to different factors other than the time factor. Hence, further studies are required to investigate the time course of depolarization induced by the quantum tunneling of lithium ions, but at this stage, the investigation is limited to the extent and degree of depolarization.

## 5. Conclusions

The present article provides a reasonable quantum tunneling model to explain the depolarizing effect of lithium ions and show the strong similarity between the effects of lithium and membrane depolarization to indicate that lithium mediates its cellular effects by membrane depolarization induced by quantum tunneling according to our calculations presented in this article.

## Figures and Tables

**Table 1:** The table shows that extracellular lithium isotopes can have a range of tunneling probability from the value at G=3 J to the value at G=7 J according to the setting values in graph (a) of Figure 2.

Lithium Isotope	$T_{Q\left(E\right)}$ at G=3 J	$T_{Q\left(E\right)}$ at G=7 J
Li-7	$2.2\times 10^{-3}$	$6.3\times 10^{-14}$
Li-6	$3.5\times 10^{-3}$	$5.8\times 10^{-13}$

**Table 2:** The table shows that extracellular lithium isotopes can have a range of tunneling probability from the value at L=0 m to the value at L=2 m according to the setting values in graph (b) of Figure 2.

Lithium Isotope	$T_{Q\left(E\right)}\;\mathrm{at}\;L=0\;m$	$T_{Q\left(E\right)}\;\mathrm{at}\;L=2\;m$
Li-7	1	$8.5\times 10^{-18}$
Li-6	1	$1.5\times 10^{-16}$

**Table 3:** The table shows that extracellular lithium isotopes can have a range of tunneling probability from the value at $V_{m}=0$ V to the value at $V_{m}=0.087$ V according to the setting values in graph (c) of Figure 2.

Lithium Isotope	$T_{Q\left(E\right)}$ $\;\mathrm{at}\;V_{m}=0\;V$	$T_{Q\left(E\right)}$ $\;\mathrm{at}\;V_{m}=0.087\;V$
Li-7	$6.6\times 10^{-16}$	$2.97\times 10^{-8}$
Li-6	$8.4\times 10^{-15}$	$1.2\times 10^{-8}$

**Table 4:** The table shows that intracellular lithium isotopes can have a range of tunneling probability from the value at G=3 J to the value at G=7 J according to the setting values in graph (a) of Figure 3.

Lithium Isotope	$T_{Q\left(I\right)}$ at G=3 J	$T_{Q\left(I\right)}$ at G=7 J
Li-7	$8.4\times 10^{-11}$	$7.8\times 10^{-20}$
Li-6	$4.5\times 10^{-10}$	$1.9\times 10^{-18}$

**Table 5:** The table shows that intracellular lithium isotopes can have a range of tunneling probability from the value at L=0 m to the value at L=2 m according to the setting values in graph (b) of Figure 3.

Lithium Isotope	$T_{Q\left(I\right)}$ at L=0 m	$T_{Q\left(I\right)}$ at L=2 m
Li-7	1	$4.3\times 10^{-31}$
Li-6	1	$7.1\times 10^{-29}$

**Table 6:** The table shows that extracellular lithium isotopes can have a range of quantum conductance of single channel from the value at G=3 J to the value at G=7 J according to the setting values in graph (a) of Figure 4.

Lithium Isotope	$C_{Q\left(E\right)}$ at G=3 J	$C_{Q\left(E\right)}$ at G=7 J
Li-7	$8.6\times 10^{-8}$ S	$2.5\times 10^{-18}$ S
Li-6	$1.3\times 10^{-7}$ S	$2.3\times 10^{-17}$ S

**Table 7:** The table shows that extracellular lithium isotopes can have a range of quantum conductance of single channel from the value at L=0 m to the value at L=2  m according to the setting values in graph (b) of Figure 4.

Lithium Isotope	$C_{Q\left(E\right)}$ at L=0 m	$C_{Q\left(E\right)}$ at L=2 m
Li-7	$3.9\times 10^{-5}$ S	$3.3\times 10^{-22}$
Li-6	$3.9\times 10^{-5}$ S	$5.8\times 10^{-21}$

**Table 8:** The table shows that extracellular lithium isotopes can have a range of quantum conductance of single channel from the value at $V_{m}=0$ V to the value at $V_{m}=0.087$ V according to the setting values in graph (c) of Figure 4.

Lithium Isotope	$C_{Q\left(E\right)}$ $\;\mathrm{at}\;V_{m}=0\;V$	$C_{Q\left(E\right)}$ $\;\mathrm{at}\;V_{m}=0.087\;V$
Li-7	$2.6\times 10^{-20}$ S	$1.2\times 10^{-13}$ S
Li-6	$3.3\times 10^{-19}$ S	$4.8\times 10^{-13}$ S

**Table 9:** The table shows that intracellular lithium isotopes can have a range of quantum conductance of single channel from the value at G=3 J to the value at G=7 J according to the setting values in graph (a) of Figure 5.

Lithium Isotope	$C_{Q\left(I\right)}$ at G=3 J	$C_{Q\left(I\right)}$ at G=7 J
Li-7	$3.3\times 10^{-15}$ S	$3\times 10^{-24}$ S
Li-6	$1.8\times 10^{-14}$ S	$7.5\times 10^{-23}$ S

**Table 10:** The table shows that intracellular lithium isotopes can have a range of quantum conductance of single channel from the value at L=0 m to the value at L=2  J according to the setting values in graph (b) of Figure 5.

Lithium Isotope	$C_{Q\left(I\right)}$ at L=0 m	$C_{Q\left(I\right)}$ at L=2 m
Li-7	$3.9\times 10^{-5}$ S	$1.7\times 10^{-35}$ S
Li-6	$3.9\times 10^{-5}$ S	$2.7\times 10^{-33}$ S

**Table 11:** The table shows that extracellular lithium isotopes can have a range of quantum membrane conductance from the value at G=3 J to the value at G=7 J according to the setting values in graph (a) of Figure 6.

Lithium Isotope	$MC_{Q\left(E\right)}$ at G=3 J	$MC_{Q\left(E\right)}$ at G=7 J
Li-7	$8.6\times 10^{6}$ mS/cm^2^	$2.5\times 10^{-4}$ mS/cm^2^
Li-6	$1.3\times 10^{7}$ mS/cm^2^	$2.3\times 10^{-3}$ mS/cm^2^

**Table 12:** The table shows that extracellular lithium isotopes can have a range of quantum membrane conductance from the value at L=0 m to the value at L=2 m according to the setting values in graph (b) of Figure 6.

Lithium Isotope	$MC_{Q\left(E\right)}$ at L=0 m	$MC_{Q\left(E\right)}$ at L=2 m
Li-7	$3.9\times 10^{9}$ mS/cm^2^	$3.3\times 10^{-8}$ mS/cm^2^
Li-6	$3.9\times 10^{9}$ mS/cm^2^	$5.8\times 10^{-7}$ mS/cm^2^

**Table 13:** The table shows that extracellular lithium isotopes can have a range of quantum membrane conductance from the value at $V_{m}=0$ V to the value at $V_{m}=0.087$ V according to the setting values in graph (c) of Figure 6.

Lithium Isotope	$MC_{Q\left(E\right)}$ $\;\mathrm{at}\;V_{m}=0\;m$	$MC_{Q\left(E\right)}$ $\;\mathrm{at}\;V_{m}=0.087\;V$
Li-7	$2.5\times 10^{-6}$ mS/cm^2^	11.5 mS/cm^2^
Li-6	$3.3\times 10^{-5}$ mS/cm^2^	48.2 mS/cm^2^

**Table 14:** The table shows that extracellular lithium isotopes can have a range of quantum membrane conductance from the value at $D=10^{9}$ channels/cm^2^ to the value at $D=10^{11}$ channels/cm^2^ according to the setting values in graph (d) of Figure 6.

Lithium Isotope	$MC_{Q\left(E\right)}$ $\;\mathrm{at}\;D=10^{9}\mathrm{channels}/{\mathrm{cm}}^{2}$	$MC_{Q\left(E\right)}$ $\;\mathrm{at}\;D=10^{11}\mathrm{channels}/{\mathrm{cm}}^{2}$
Li-7	0.11 mS/cm^2^	11.3 mS/cm^2^
Li-6	0.47 mS/cm^2^	47.4 mS/cm^2^

**Table 15:** The table shows that intracellular lithium isotopes can have a range of quantum membrane conductance from the value at G=3 J to the value at G=7 J according to the setting values in graph (a) of Figure 7.

Lithium Isotope	$MC_{Q\left(I\right)}$ at G=3 J	$MC_{Q\left(I\right)}$ at G=7 J
Li-7	0.33 mS/cm^2^	$3\times 10^{-10}$ mS/cm^2^
Li-6	1.76 mS/cm^2^	$7.5\times 10^{-9}$ mS/cm^2^

**Table 16:** The table shows that intracellular lithium isotopes can have a range of quantum membrane conductance from the value at L=0 m to the value at L=2 m according to the setting values in graph (b) of Figure 7.

Lithium Isotope	$MC_{Q\left(I\right)}$ at L=0 m	$MC_{Q\left(I\right)}$ at L=2 m
Li-7	$3.9\times 10^{9}$ mS/cm^2^	$1.7\times 10^{-21}$ mS/cm^2^
Li-6	$3.9\times 10^{9}$ mS/cm^2^	$2.7\times 10^{-19}$ mS/cm^2^

**Table 17:** The table shows that intracellular lithium isotopes can have a range of quantum membrane conductance from the value at $D=10^{9}$ channels/cm^2^ to the value at $D=10^{11}$ channels/cm^2^ according to the setting values in graph (c) of Figure 7.

Lithium Isotope	$MC_{Q\left(I\right)}$ $\;\mathrm{at}\;D=10^{9}\mathrm{channels}/{\mathrm{cm}}^{2}$	$MC_{Q\left(I\right)}$ $\;\mathrm{at}\;D=10^{11}\mathrm{channels}/{\mathrm{cm}}^{2}$
Li-7	$2.6\times 10^{-8}$ mS/cm^2^	$2.6\times 10^{-6}$ mS/cm^2^
Li-6	$3.3\times 10^{-7}$ mS/cm^2^	$3.3\times 10^{-5}$ mS/cm^2^

**Table 18:** The table shows the membrane potential values at extracellular lithium concentration of 1 mmol/L and 100 mmol/L, at different concentration ratios R and based on the classical transport of both isotopes as in graph (a) of Figure 8.

The Concentration Ratio R	$V_{m}$ $\;\mathrm{at}\;{\left[Li\right]}_{E}=1\;\mathrm{mmol}/L$	$V_{m}$ $\;\mathrm{at}\;{\left[Li\right]}_{E}=100\;\mathrm{mmol}/L$
1	0.087 V	0.083 V
2	0.087 V	0.083 V
3	0.087 V	0.083 V
4	0.087 V	0.083 V

**Table 19:** The table shows the depolarization degree based on the classical transport of both isotopes as in graph (a) of Figure 8.

The Concentration Ratio R	$\mathrm{Depolarization}\;\mathrm{Degree}\;\mathrm{at}\;{\left[Li\right]}_{E}=1\;\mathrm{mmol}/L$	$\mathrm{Depolarization}\;\mathrm{Degree}\;\mathrm{at}\;{\left[Li\right]}_{E}=100\;\mathrm{mmol}/L$
1	0 V	0.004 V
2	0 V	0.004 V
3	0 V	0.004 V
4	0 V	0.004 V

**Table 20:** The table shows the membrane potential values at extracellular lithium concentration of 1 mmol/L and 100  mmol/L, at different concentration ratios R, based on the classical transport of both isotopes as in graph (b) of Figure 8.

The Concentration Ratio R	$V_{m}$ $\;\mathrm{at}\;{\left[Li\right]}_{E}=1\;\mathrm{mmol}/L$	$V_{m}$ $\;\mathrm{at}\;{\left[Li\right]}_{E}=100\;\mathrm{mmol}/L$
1	0.055 V	0.045 V
2	0.055 V	0.047 V
3	0.055 V	0.048 V
4	0.055 V	0.05 V

**Table 21:** The table shows the depolarization degree based on the classical transport of both isotopes as in graph (b) of Figure 8.

The Concentration Ratio R	$\mathrm{Depolarization}\;\mathrm{Degree}\;\mathrm{at}\;{\left[Li\right]}_{E}=1\;\mathrm{mmol}/L$	$\mathrm{Depolarization}\;\mathrm{Degree}\;\mathrm{at}\;{\left[Li\right]}_{E}=100\;\mathrm{mmol}/L$
1	0 V	0.01 V
2	0 V	0.008 V
3	0 V	0.007 V
4	0 V	0.005 V

**Table 22:** The table shows the membrane potential values at extracellular lithium concentration of 1 mmol/L and 100  mmol/L, at different concentration ratios R and based on the classical transport of Li-6 as in graph (a) of Figure 9.

The Concentration Ratio R	$V_{m}$ $\;\mathrm{at}\;{\left[Li\right]}_{E}=1\;\mathrm{mmol}/L$	$V_{m}$ $\;\mathrm{at}\;{\left[Li\right]}_{E}=100\;\mathrm{mmol}/L$
1	0.087 V	0.082 V
2	0.087 V	0.082 V
3	0.087 V	0.082 V
4	0.087 V	0.082 V

**Table 23:** The table shows the depolarization degree based on the classical transport of Li-6 as in graph (a) of Figure 9.

The Concentration Ratio R	$\mathrm{Depolarization}\;\mathrm{Degree}\;\mathrm{at}\;{\left[Li\right]}_{E}=1\;\mathrm{mmol}/L$	$\mathrm{Depolarization}\;\mathrm{Degree}\;\mathrm{at}\;{\left[Li\right]}_{E}=100\;\mathrm{mmol}/L$
1	0 V	0.005 V
2	0 V	0.005 V
3	0 V	0.005 V
4	0 V	0.005 V

**Table 24:** The table shows the membrane potential values at extracellular lithium concentration of 1 mmol/L and 100  mmol/L, at different concentration ratios R and based on the classical transport of Li-6 as in graph (b) of Figure 9.

The Concentration Ratio R	$V_{m}$ $\;\mathrm{at}\;{\left[Li\right]}_{E}=1\;\mathrm{mmol}/L$	$V_{m}$ $\;\mathrm{at}\;{\left[Li\right]}_{E}=100\;\mathrm{mmol}/L$
1	0.055 V	0.044 V
2	0.055 V	0.046 V
3	0.055 V	0.047 V
4	0.055 V	0.049 V

**Table 25:** The table shows the depolarization degree based on the classical transport of Li-6 as in graph (b) of Figure 9.

The Concentration Ratio R	$\mathrm{Depolarization}\;\mathrm{Degree}\;\mathrm{at}\;{\left[Li\right]}_{E}=1\;\mathrm{mmol}/L$	$\mathrm{Depolarization}\;\mathrm{Degree}\;\mathrm{at}\;{\left[Li\right]}_{E}=100\;\mathrm{mmol}/L$
1	0 V	0.011 V
2	0 V	0.009 V
3	0 V	0.008 V
4	0 V	0.006 V

**Table 26:** The table shows the membrane potential values at extracellular lithium concentration of 1 mmol/L and 100 mmol/L and based on the quantum transport and the corresponding setting values as in graph (a) of Figure 10.

Lithium Isotope	$V_{m}$ $\;\mathrm{at}\;{\left[Li\right]}_{E}=1\;\mathrm{mmol}/L$	$V_{m}$ $\;\mathrm{at}\;{\left[Li\right]}_{E}=100\;\mathrm{mmol}/L$
Li-7	0.075 V	0.055 V
Li-6	0.068 V	0.047 V

**Table 27:** The table shows the depolarization degree based on the quantum transport of both isotopes as in graph (a) of Figure 10.

Lithium Isotope	$\mathrm{Depolarization}\;\mathrm{Degree}\;\mathrm{at}\;{\left[Li\right]}_{E}=1\;\mathrm{mmol}/L$	$\mathrm{Depolarization}\;\mathrm{Degree}\;\mathrm{at}\;{\left[Li\right]}_{E}=100\;\mathrm{mmol}/L$
Li-7	0.012 V	0.032 V
Li-6	0.019 V	0.04 V

**Table 28:** The table shows the membrane potential values at extracellular lithium concentration of 1 mmol/L and 100 mmol/L and based on the quantum transport and the corresponding setting values as in graph (b) of Figure 10.

Lithium Isotope	$V_{m}$ $\;\mathrm{at}\;{\left[Li\right]}_{E}=1\;\mathrm{mmol}/L$	$V_{m}$ $\;\mathrm{at}\;{\left[Li\right]}_{E}=100\;\mathrm{mmol}/L$
Li-7	0.082 V	0.065 V
Li-6	0.077 V	0.058 V

**Table 29:** The table shows the depolarization degree based on the quantum transport of lithium isotopes as in graph (b) of Figure 10.

Lithium Isotope	$\mathrm{Depolarization}\;\mathrm{Degree}\;\mathrm{at}\;{\left[Li\right]}_{E}=1\;\mathrm{mmol}/L$	$\mathrm{Depolarization}\;\mathrm{Degree}\;\mathrm{at}\;{\left[Li\right]}_{E}=100\;\mathrm{mmol}/L$
Li-7	0.005 V	0.022 V
Li-6	0.01 V	0.029 V

**Table 30:** The table shows the membrane potential values at extracellular lithium concentration of 1 mmol/L and 100 mmol/L and based on the quantum transport and the corresponding setting values as in graph (c) of Figure 10.

Lithium Isotope	$V_{m}$ $\;\mathrm{at}\;{\left[Li\right]}_{E}=1\;\mathrm{mmol}/L$	$V_{m}$ $\;\mathrm{at}\;{\left[Li\right]}_{E}=100\;\mathrm{mmol}/L$
Li-7	0.086 V	0.075 V
Li-6	0.084 V	0.068 V

**Table 31:** The table shows the depolarization degree based on the quantum transport of both isotopes as in graph (c) of Figure 10.

Lithium Isotope	$\mathrm{Degree}\;\mathrm{of}\;\mathrm{Depolarization}\;\mathrm{at}\;{\left[Li\right]}_{E}=1\;\mathrm{mmol}/L$	$\mathrm{Degree}\;\mathrm{of}\;\mathrm{Depolarization}\;\mathrm{at}\;{\left[Li\right]}_{E}=100\;\mathrm{mmol}/L$
Li-7	0.001 V	0.012 V
Li-6	0.003 V	0.019 V

**Table 32:** The table shows the ratio Li-6/Li-7 (*E*) that can take the range between the two values at G=3 J and at G=7 J and shows the rate of change in the ratio with respect to the energy of gate *G*.

Li-6/Li-7 (E) at G=3 J	Li-6/Li-7 (E) at G=7 J	Rate of Change *A(G)*
1.56	9.17	1.9 J^−1^

**Table 33:** The table shows the ratio Li-6/Li-7 (*E*) that can take the range between the two values at L=0 m and at L=2 m and shows the rate of change in the ratio with respect to the length of gate *L*.

Li-6/Li-7 (E) at L=0 m	Li-6/Li-7 (E) at L=2 m	Rate of Change *A(L)*
1	17.57	8.29 m^−1^

**Table 34:** The table shows the ratio Li-6/Li-7 (*E*) that can take the range between the two values at $V_{m}=0$ V and at $V_{m}=0.087$ V and shows the rate of change in the ratio with respect to the membrane potential.

$\mathrm{Li}-6/\mathrm{Li}-7\;(E)\;\mathrm{at}\;V_{m}=0\;V$	$\mathrm{Li}-6/\mathrm{Li}-7\;(E)\;\mathrm{at}\;V_{m}=0.087\;V$	Rate of Change *A(V_m_)*
12.8	4.19	−0.1 (mV)^−1^

**Table 35:** The table shows the ratio Li-6/Li-7 (*I*) that can take the range between the two values at G=3 J and at G=7 J and shows the rate of change in the ratio with respect to the energy of gate *G*.

Li-6/Li-7 (I) at G=3 J	Li-6/Li-7 (I) at G=7 J	Rate of Change *A (G)*
5.43	24.73	4.83 J^−1^

**Table 36:** The table shows the ratio Li-6/Li-7 *(I*) that can take the range between the two values at L=0 m and at L=2 m and shows the rate of change in the ratio with respect to the length of gate *L*.

Li-6/Li-7 (I) at L=0 m	Li-6/Li-7 (I) at L=2 m	Rate of Change A(L)
1	163.73	81.37 m^−1^

## Data Availability

Not applicable.
